# Variations in candidalysin amino acid sequence influence toxicity and host responses

**DOI:** 10.1128/mbio.03351-23

**Published:** 2024-07-02

**Authors:** Don N. Wickramasinghe, Claire M. Lyon, Sejeong Lee, Olivia W. Hepworth, Emily L. Priest, Corinne Maufrais, Adam P. Ryan, Emmanuelle Permal, Derek Sullivan, Brenda A. McManus, Bernhard Hube, Geraldine Butler, Christophe d'Enfert, Julian R. Naglik, Jonathan P. Richardson

**Affiliations:** 1 Centre for Host-Microbiome Interactions, Faculty of Dentistry, Oral & Craniofacial Sciences, King’s College London, London, United Kingdom; 2 Institut Pasteur, Université Paris Cité, INRAe USC 2019, Unité Biologie et Pathogénicité Fongiques, Paris, France; 3 Institut Pasteur, Université Paris Cité, Bioinformatics and Biostatistics Hub, Paris, France; 4 School of Biomedical and Biomolecular Science and UCD Conway Institute of Biomolecular and Biomedical Research, Conway Institute, University College Dublin, Dublin, Ireland; 5 Division of Oral Biosciences, Dublin Dental University Hospital, and School of Dental Science, Trinity College Dublin, Dublin, Ireland; 6 Department of Microbial Pathogenicity Mechanisms, Leibniz Institute for Natural Product Research and Infection Biology, Hans Knoll Institute (HKI), Jena, Germany; 7 Institute of Microbiology, Friedrich Schiller University, Jena, Germany; The University of Texas Health Science Center at Houston, Houston, Texas, USA

**Keywords:** *Candida*, candidalysin, fungus, toxin, epithelial

## Abstract

*Candida albicans* causes millions of mucosal infections in humans annually. Hyphal overgrowth on mucosal surfaces is frequently associated with tissue damage caused by candidalysin, a secreted peptide toxin that destabilizes the plasma membrane of host cells thereby promoting disease and immunopathology. Candidalysin was first identified in *C. albicans* strain SC5314, but recent investigations have revealed candidalysin “variants” of differing amino acid sequence in isolates of *C. albicans*, and the related species *C. dubliniensis*, and *C tropicalis*, suggesting that sequence variation among candidalysins may be widespread in natural populations of these *Candida* species. Here, we analyzed *ECE1* gene sequences from 182 *C*. *albicans* isolates, 10 *C*. *dubliniensis* isolates, and 78 *C*. *tropicalis* isolates and identified 10, 3, and 2 candidalysin variants in these species, respectively. Application of candidalysin variants to epithelial cells revealed differences in the ability to cause cellular damage, changes in metabolic activity, calcium influx, MAPK signalling, and cytokine secretion, while biophysical analyses indicated that variants exhibited differences in their ability to interact with and permeabilize a membrane. This study identifies candidalysin variants with differences in biological activity that are present in medically relevant *Candida* species.

**IMPORTANCE:**

Fungal infections are a significant burden to health. Candidalysin is a toxin produced by *Candida albicans* that damages host tissues, facilitating infection. Previously, we demonstrated that candidalysins exist in the related species *C. dubliniensis* and *C. tropicalis*, thereby identifying these molecules as a toxin family. Recent genomic analyses have highlighted the presence of a small number of candidalysin “variant” toxins, which have different amino acid sequences to those originally identified. Here, we screened genome sequences of isolates of *C. albicans*, *C. dubliniensis*, and *C. tropicalis* and identified candidalysin variants in all three species. When applied to epithelial cells, candidalysin variants differed in their ability to cause damage, activate intracellular signaling pathways, and induce innate immune responses, while biophysical analysis revealed differences in the ability of candidalysin variants to interact with lipid bilayers. These findings suggest that intraspecies variation in candidalysin amino acid sequence may influence fungal pathogenicity.

## INTRODUCTION

The incidence of fungal infections has increased dramatically in recent years and is a major driver of morbidity and mortality in humans (1). *Candida* species cause superficial infections such as mucosal candidiasis, and life-threatening systemic infections in susceptible individuals. *Candida albicans* normally colonizes the mucosal surfaces of the human body as a commensal organism, but can, under suitably predisposing conditions, overgrow at mucosal surfaces and cause disease.

A defining feature of *C. albicans* pathogenicity at mucosal surfaces is the ability to transition from ovoid yeasts to invasive hyphae (2), a process underpinned by robust changes in gene expression (3). Hypha formation enables *C. albicans* to adhere strongly to mucosal surfaces and invade epithelial cells by active penetration or receptor-induced endocytosis (4–6). Invasion of *C. albicans* is accompanied by the formation of an invasion “pocket” into which the cytolytic toxin candidalysin is secreted (7, 8). Candidalysin is generated from the hypha-specific polyprotein extent of elongation 1 (Ece1p) (9), which is cleaved sequentially, and in a context-dependent manner (10), by the Golgi-associated proteases Kex2p and Kex1p (7, 11, 12). Once released from Ece1p, candidalysin is secreted from the invading hypha into the extracellular environment of the invasion pocket (8, 13) where it damages host cells by permeabilizing epithelial plasma membranes (7), induces rapid mitochondrial shutdown, and necrotic cell death (14).

Candidalysin drives innate epithelial immune responses through two nonredundant signaling axes involving EGFR-ERK1/2-c-Fos and MKK3/6-p38, which regulate the secretion of granulocyte-colony-stimulating factor (G-CSF), granulocyte macrophage-colony-stimulating factor (GM-CSF), and interleukin (IL)−6, respectively (15). The secretion of cytokines and other alarmins (16) from infected epithelial cells serves to recruit and activate host immune cells, which are critical for the resolution of infections.

Candidalysin toxins are found in *C. albicans,* but also in *C. dubliniensis* and *C. tropicalis*, and exhibit sequence variation at the amino acid level (12, 17, 18). The discovery of the candidalysin family (17) combined with candidalysin sequence variation between different strains of *C. albicans* (12, 18) suggests that candidalysin sequence variation within populations of *Candida* fungi may be a widespread phenomenon. To better understand the variation in candidalysin sequence within natural populations of *Candida* species, we analyzd *ECE1* gene sequences from isolates of *C. albicans*, *C. dubliniensis*, and *C. tropicalis*. In addition to the previously characterized candidalysins, we identified eight new candidalysin variant sequences in *C. albicans*, two in *C. dubliniensis*, and one in *C. tropicalis*. Biophysical and biological analysis of these new candidalysin variants revealed differences in toxicity and epithelial activation *in vitro*. This study identifies widespread sequence variation in the candidalysins of *C. albicans*, but not *C. dubliniensis* or *C. tropicalis*, and demonstrates that candidalysins possess different potencies that may influence fungal pathogenicity.

## RESULTS

### Identification of naturally occurring candidalysin variants in *C. albicans*, *C. dubliniensis*, and *C. tropicalis*

To explore variations in candidalysin sequences, we mapped single-nucleotide polymorphisms (SNPs) across the region of the *ECE1* gene encoding candidalysin in 182 isolates of *C. albicans* (19), 10 isolates of *C. dubliniensis* (Table S1), and 78 isolates of *C. tropicalis* (20). Haplotypes were determined (see Materials and Methods), and the amino acid sequence of candidalysin was inferred for each haplotype (21).

We identified 10 variants of *C. albicans* candidalysin (designated A–J; Table 1) from 364 *ECE1* alleles, including the previously characterized candidalysin of the hypervirulent strain SC5314 (7) (referred to herein as variant A), and the commensal isolate 529 L (12, 22) (referred to herein as variant J). The most common candidalysin variant identified was F(115/364) > H(100/364) > A(66/364) > J(41/364) > G(24/364) > I(11/364) > D(2/364) = E(2/364) > B(1/364) = C(1/364). The most common homozygous variant pairing was H(44/182) > F(36/182) > A(14/182) > J(13/182) > I(5/182) > G(4/182) > E(1/182) > B + C + D(0/182), while the most common variant pairings in heterozygous isolates of *C. albicans* were A + F (24/182) and F + G (14/182) (Fig. S1).

**TABLE 1:** Candidalysin sequence variation in isolates of *C. albicans*, *C. dubliniensis*, and *C. tropicalis^a^*

Candidalysin	Amino acid sequence	Frequency
*C. albicans*		364
SC5314 Clys—Variant A	SIIGIIMGILGNIPQVIQIIMSIVKAFKGNK	67
*C. alb* Clys—Variant B	SII**S**IIMGILGNIPQVIQIIMSIVKAFKGNK	1
*C. alb* Clys—Variant C	SIIGIIMGIL**T**NIPQVIQIIMSIVKAFKGNK	1
*C. alb* Clys—Variant D	SIIGIIMGILGNIPQVIQIIMSIV**R**AFKGNK	2
*C. alb* Clys—Variant E	SII**S**IIMG**L**LGNIPQVIQIIMSIVKAFKGNK	2
*C. alb* Clys—Variant F	SII**S**IIMGIL**T**NIPQVIQIIMSIV**R**AFKGNK	115
*C. alb* Clys—Variant G	SII**S**IIMGIL**S**NIPQVIQIIMSIV**R**AFKGNK	24
*C. alb* Clys—Variant H	SIIGIIMG**L**L**T**NIPQVIQIIMSIV**R**AFKGNK	100
*C. alb* Clys—Variant I	SIIGIIMG**L**L**T**NIPQVI**N**IIMSI**I**KAFKGNK	11
*C. alb* Clys—Variant J	S**FLS**II**TAL**LGNIPQ**I**IQIIM**G**IVKAF**R**GNK	41
*C. dubliniensis*		20
CD36 Clys—Variant A	SIIGILTAILNNVPQIINVITTIIKSITGNK	12
*C. dub* Clys—Variant B	SIIGILTAILNN**I**PQIINVITTIIKSITGNK	4
*C. dub* Clys—Variant C	SIIGILTAILNNVPQIINVI**M**TIIKSITGNK	4
*C. tropicalis*		156
MYA-3404 Clys—Variant A	ISFAGIVSSIINQLPSIIQIIGNIIKAGLVK	150
*C. trop* Clys—Variant B	**L**SFAGIV**G**SIINQLPSIIQIIGNIIKAGLVK	6

^*a*^ Variations in amino acid sequence are indicated (boldface and underlined). The allelic frequency is indicated (total number of alleles for each candidalysin variant).

We identified three variants of *C. dubliniensis* candidalysin (designated A–C; Table 1) from 20 *ECE1* alleles, including the previously characterized candidalysin from reference strain CD36 (referred to herein as variant A) (17). The most common candidalysin variant identified across all sequenced alleles was A(12/20) > B(4/20) = C(4/20). The most common homozygous variant pairing was A(6/10) > B(2/10) = C(2/10). None of the isolates characterized possessed two distinct candidalysin variants (Fig. S2).

We also identified two variants of *C. tropicalis* candidalysin (designated A and B; Table 1) from 156 alleles of the *ECE1* ortholog *CTRG_00476*, including the previously characterized candidalysin from reference strain MYA-3404 (referred to herein as variant A) (17). The most common candidalysin variant identified across all sequenced alleles was A(150/156) > B(6/156). The most common homozygous variant pairing was A(72/78), while six isolates encoded both variants A and B (Fig. S3).

Collectively, these data suggest that candidalysin sequence variation exists within populations of *Candida* species. Having identified sequence variation in the candidalysins of *C. albicans*, *C. dubliniensis*, and *C. tropicalis*, peptides corresponding to these sequences (Table 1) were synthesized and their biophysical and biological properties were compared *in vitro*.

### Candidalysin variants are α-helical and amphipathic

**Fig 1:**
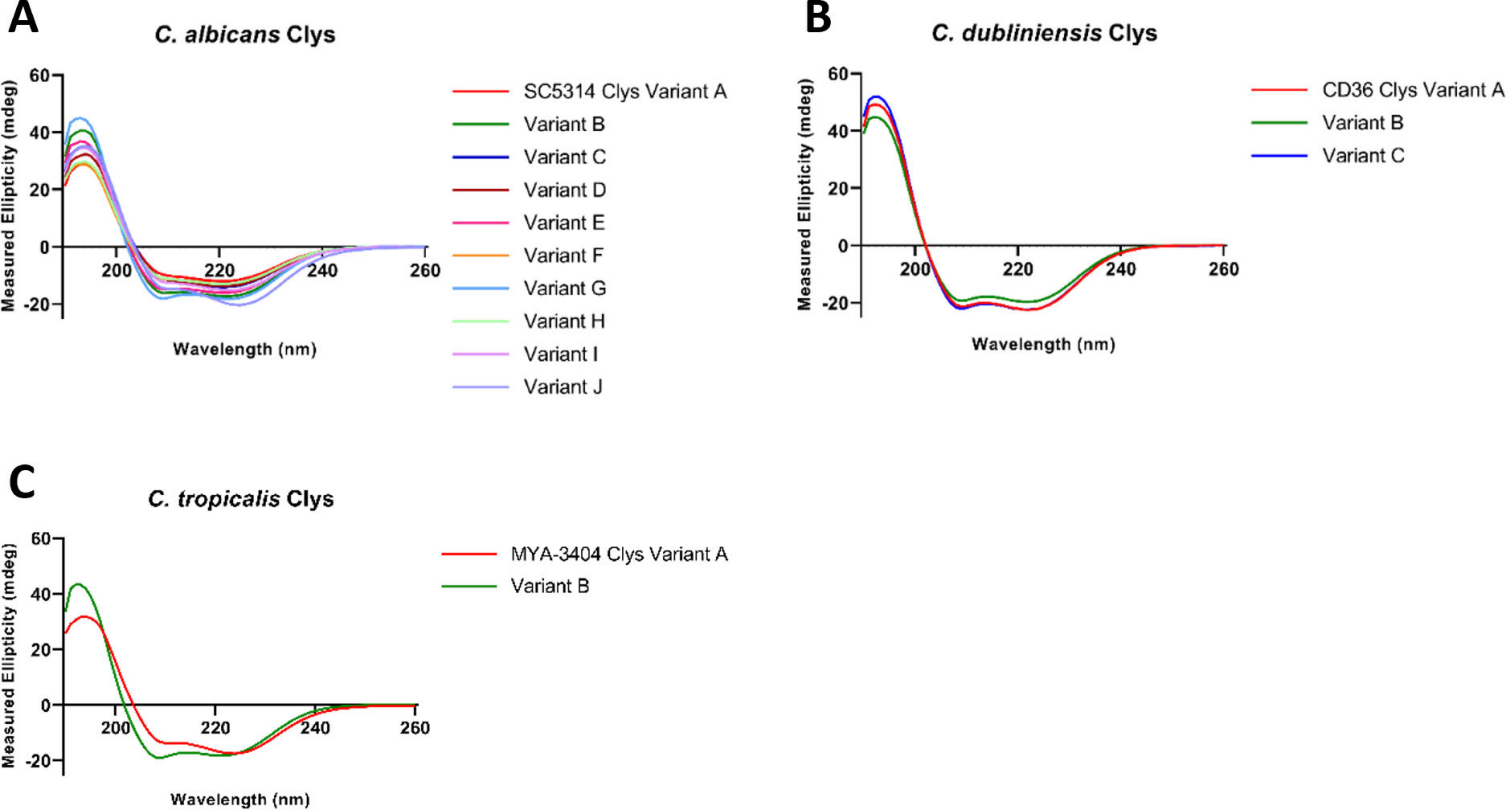
Candidalysin variants adopt an α-helical conformation in solution. Circular dichroism spectroscopy of candidalysin (Clys) variants from (**A**) *C. albicans*, (**B**) *C. dubliniensis*, and (**C**) *C. tropicalis*. Data are the mean of *n* = 3 biological repeats.

Candidalysins from *C. albicans* SC5314, *C. dubliniensis* CD36, and *C. tropicalis* MYA-3404 adopt a predominantly α-helical secondary structure in aqueous solution and present an asymmetric distribution of surface charge along the length of the helix, consistent with amphipathic peptides (7, 17). Circular dichroism spectroscopy (Fig. 1A through C) confirmed that all of the newly identified candidalysin variants in these species were predominantly α-helical, while helical wheel renderings (23) of each candidalysin confirmed amphipathicity (Fig. S4).

### Candidalysin variants differ in their ability to cause cellular damage, rupture artificial membranes, and induce changes in metabolic activity

**Fig 2:**
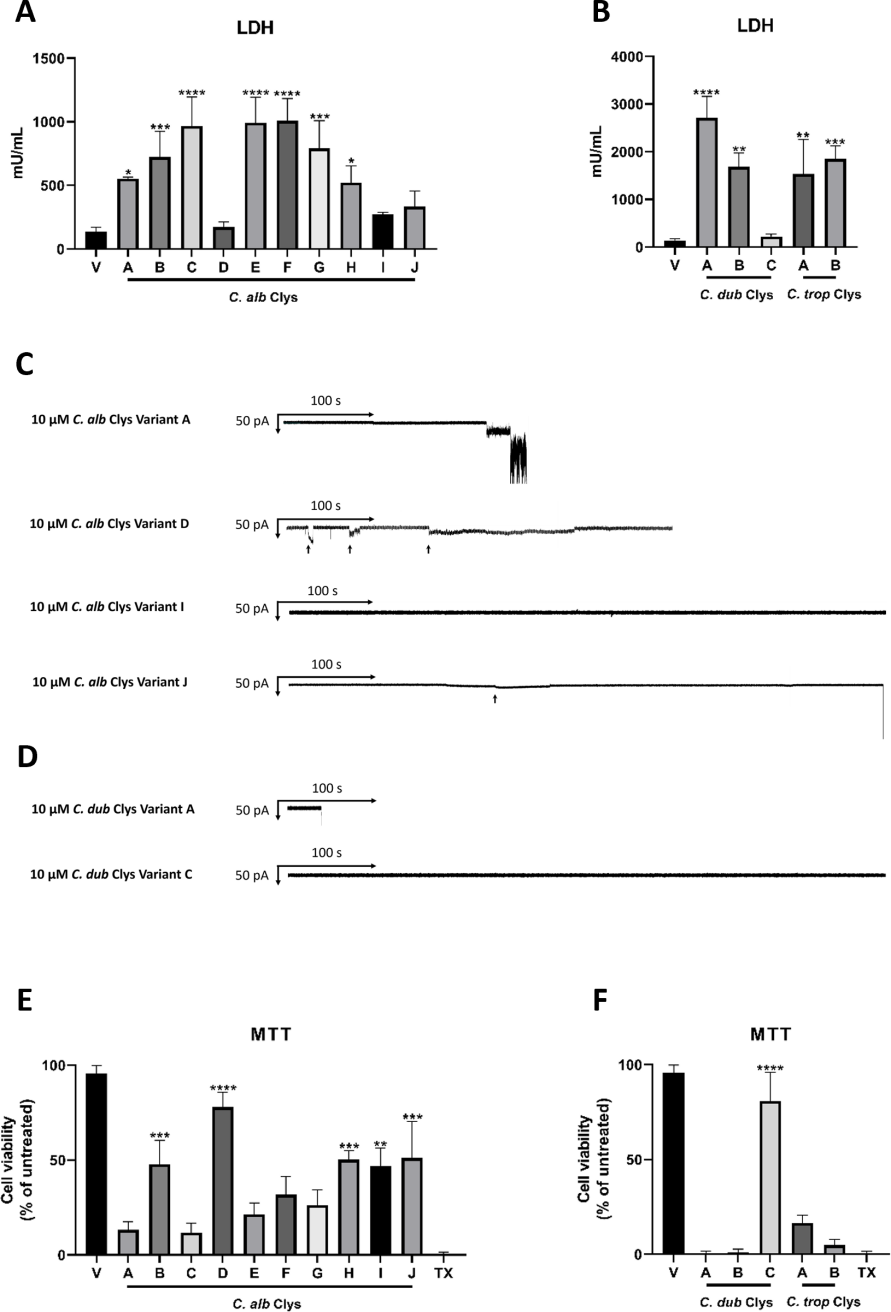
Candidalysin variants exhibit differences in their ability to cause cellular damage, membrane permeabilization and induce changes in metabolic activity. TR146 oral epithelial cells were treated with 15 µM of each candidalysin variant from (**A**) *C. albicans*, and (**B**) *C. dubliniensis* and *C. tropicalis* for 24 h, and exhausted culture medium was quantified for LDH activity. Data are the mean + SD of *n* = 3 biological repeats. Statistical analysis was applied relative to vehicle-treated cells. *C. albicans* candidalysin variant A vs; variant B, *P* = 0.6798 (not significant); variant C, *P* = 0.0220 (*); variant D, *P* = 0.0314 (*); variant E, *P* = 0.0136 (*); variant F, *P* = 0.0102 (*); and variant J, *P* = 0.4119 (not significant). *C. dubliniensis* candidalysin variant A vs variant C, *P* = <0.0001 (****). Representative traces from artificial 1,2-diphytanoyl-sn-glycero-3-phosphocholine (DPhPC) planar bilayers showing the dwell time (defined as the time elapsed between candidalysin addition and bilayer rupture) for (**C**) *C. albicans* and (**D**) *C. dubliniensis* candidalysin variants at 10 µM (black arrows indicate reversible membrane interaction events). One representative trace is presented from *n* = 7 repeats (*C. alb* A), 5 repeats (*C. alb* D), 7 repeats (*C. alb* J), and 11 repeats (*C. dub* A). *C. alb* variant I and *C. dub* variant C did not demonstrate positive membrane binding events from 48 and 44 experiments, respectively. TR146 epithelial cells were treated with 70 µM of each candidalysin variant from (**E**) *C. albicans*, and (**F**) *C. dubliniensis* and *C. tropicalis* for 6 h, and mitochondrial fitness was quantified by MTT assay. Triton X-100 (TX) was used as a positive control. Data are relative to untreated cells and presented as the mean + SD of *n* = 3 biological repeats. Statistical analysis was applied relative to the reference candidalysin (variant A)-treated cells. Statistical analysis was performed using a one-way ANOVA with a *post hoc* Bonferroni multiple comparison test; *P < 0.05, **P < 0.01, ***P < 0.001, and ****P < 0.0001.

Despite similar secondary structure, the candidalysin variants of *C. albicans* SC5314, *C. dubliniensis* CD36, and *C. tropicalis* MYA-3404 differ in potency (17). Thus, we hypothesized that the potency of the newly identified candidalysin variants may also differ. To address this, we applied candidalysin variants (15 µM and 70 µM) to TR146 oral epithelial cells for 24 h and quantified the activity of extracellular lactate dehydrogenase (LDH) as a surrogate marker of cellular damage (7, 8, 12, 17, 24–26). As observed previously (17), candidalysin from *C. albicans* SC5314, *C. dubliniensis* CD36, and *C. tropicalis* MYA-3404 induced significant levels of cellular damage when compared with vehicle-treated cells, with the candidalysins of *C. dubliniensis* and *C. tropicalis* being more potent (Fig. 2A and B; Fig. S5A and B). All variants of *C. albicans* candidalysin caused significant levels of cellular damage when compared with vehicle-treated cells except for variants D, I, and J at 15 µM, and variant D at 70 µM. Notably, variant C, E, and F caused significantly more damage than variant A (SC5314) at 15 µM, while variant D caused significantly less damage at both 15 µM and 70 µM (Fig. 2A; Fig. S5A). Similarly, all variants of *C. dubliniensis* and *C. tropicalis* candidalysin (15 µM and 70 µM) caused significant levels of cellular damage when compared to vehicle-treated cells, except for *C. dubliniensis* variant C which caused only minimal damage at 15 µM and 70 µM (Fig. 2B; Fig. S5B).

As the candidalysin variants varied in their ability to cause cellular damage, we questioned whether an additive effect on cellular damage would be observed if epithelial cells were exposed to a combination of candidalysin variants, and whether a variant that has a diminished ability to cause damage could prevent the damage caused by a damaging variant. To test this, we treated TR146 oral epithelial cells with 70 µM of *C. albicans* candidalysin variant A, D, F, G, H, I, and J alone, and with 70 µM of variant A and an equal concentration of variant D, F, G, H, I, and J for 24 h and quantified cellular damage. Variants B, C, and E were excluded from this analysis as these variants cause damage but do not occur frequently in the population of *ECE1* alleles we examined (frequencies of 1/364, 1/364, and 2/364, respectively; Table 1). None of the *C. albicans* candidalysin variant combinations tested (A + D, A + F, A + G, A + H, A + I, and A + J) had an additive or synergistic effect on damage when compared with variant A alone (Fig. S5C).

Notably, variant D caused minimal damage at a concentration of 15 µM (Fig. 2A) and significantly reduced damage at 70 µM when compared with variant A (Fig. S5A and C) but did not impair the ability of variant A to damage cells (Fig. S5C). Likewise, combining *C. dubliniensis* candidalysin variant A with variant B and C, and *C. tropicalis* candidalysin variant A with variant B had no additive or synergistic effect on damage (Fig. S5D).

Since *C. albicans* candidalysin variant D, I, and J, and *C. dubliniensis* candidalysin variant C were diminished in their ability to cause damage when applied to oral epithelial cells at a concentration of 15 µM, we analyzed these variants using Orbit e16 technology to gain a better understanding of their interaction with a bilayer membrane. Reference candidalysins and variants (10 µM) were applied to artificial 1,2-diphytanoyl-*sn*-glycero-3-phosphocholine (DPhPC) planar lipid bilayers and membrane interaction and ability to cause membrane permeabilization was monitored. Candidalysin variants D, I, and J were observed to interact differently with DPhPC bilayers: variant D exhibited frequent reversible binding events with the bilayer and transiently permeabilized the bilayer without causing permanent rupture; variant I did not interact with or permeabilize the bilayer; while variant J was observed to cause bilayer rupture but took considerably longer than candidalysin variant A (Fig. 2C). *C. dubliniensis* candidalysin variant A induced bilayer permeabilization more rapidly than *C. albicans* candidalysin variant A. In contrast, *C. dubliniensis* variant C was incapable of membrane binding or permeabilization (Fig. 2D).

Candidalysin from *C. albicans* SC5314 alters the metabolic activity of epithelial cells (14). We therefore investigated whether the candidalysin variants could induce similar changes. Epithelial cells were treated with 70 µM of each candidalysin variant for 6 h and metabolic activity was quantified by MTT assay (Fig. 2E and F). The candidalysin of *C. albicans* SC5314 (variant A) induced a marked reduction in mitochondrial fitness when compared with vehicle-treated cells (Fig. 2E). *C. albicans* candidalysin variant C, E, F, and G also caused similar levels of metabolic activity as SC5314 candidalysin. In contrast, variant B, D, H, I, and J permitted significantly more metabolic activity than SC5314 candidalysin (Fig. 2E). Similarly, all variants of *C. dubliniensis* and *C. tropicalis* candidalysin caused a robust reduction in mitochondrial fitness except for *C. dubliniensis* variant C (Fig. 2F). We reported previously that high concentrations (70 uM) of candidalysin are associated with cellular damage and necrosis (6, 11). Therefore, to rule out concentration-dependent effects that could be associated with candidalysin toxicity, we repeated the analysis of epithelial metabolic activity with a lower concentration (15 µM) of each candidalysin variant. At this reduced concentration, *C. albicans* candidalysin variant A exhibited a non-significant but nevertheless marked reduction in metabolic activity, as did candidalysin variant C and F. Similarly, all variants of *C. dubliniensis* and *C. tropicalis* candidalysin caused a robust reduction in mitochondrial fitness except for *C. dubliniensis* variant C (Fig. S6).

Collectively, these data indicate that candidalysin variants found in *C. albicans* and *C. dubliniensis*, but not *C. tropicalis*, exhibit differences in their ability to damage epithelial cells, rupture artificial membrane bilayers, and induce changes in metabolic activity.

### Candidalysin variants differ in their ability to induce calcium influx

**Fig 3:**
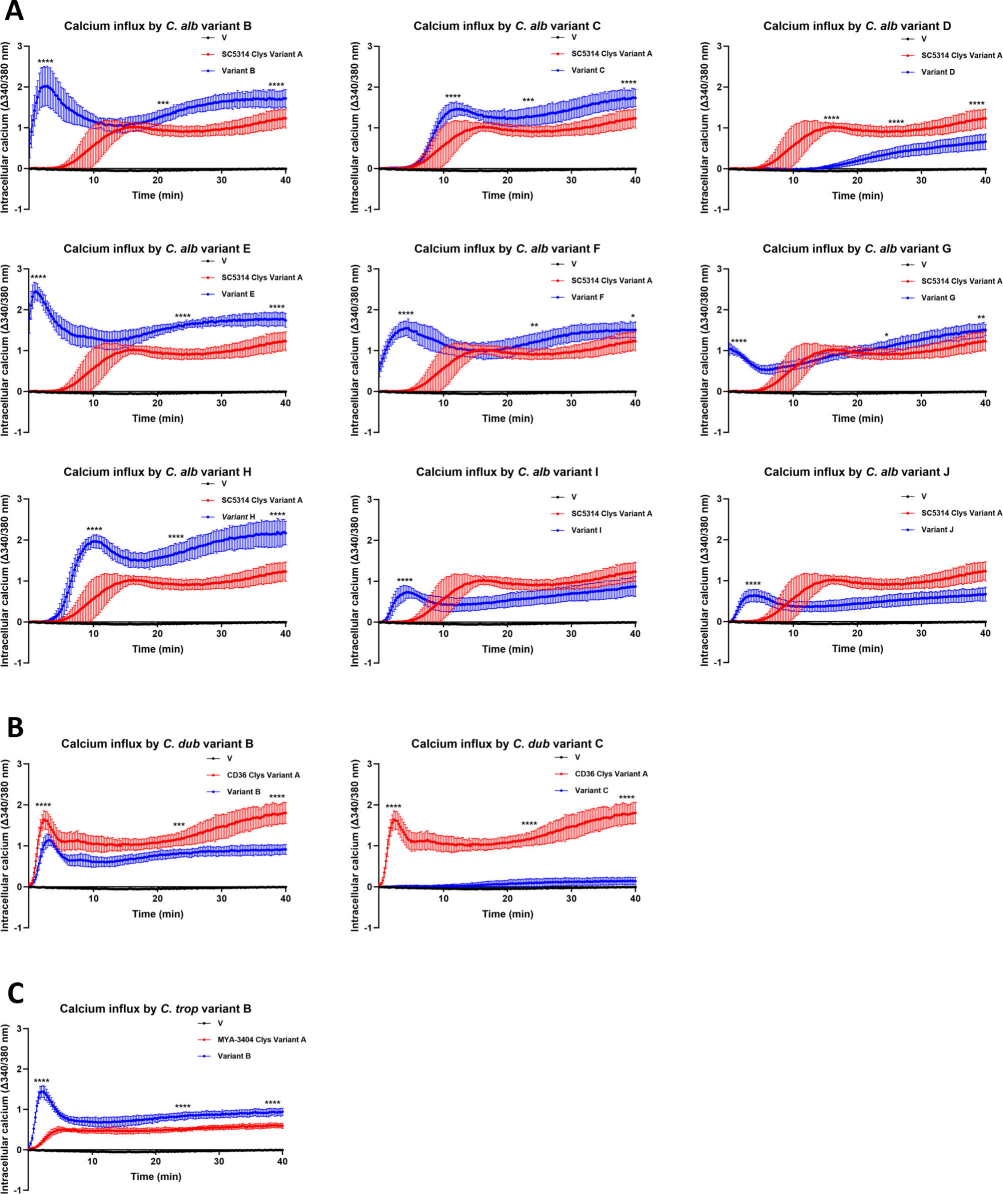
Candidalysin variants differ in their ability to induce calcium influx. TR146 oral epithelial cells were treated with 70 µM of each candidalysin variant from (**A**) *C. albicans*, (**B**) *C. dubliniensis*, and (**C**) *C. tropicalis* for 40 min, and calcium influx was quantified (Black: vehicle-treated cells, Red: reference candidalysin-treated cells, Blue: candidalysin variant-treated cells). Data are the mean + SD from *n* = 3 biological repeats. Statistical analysis was applied at the indicated time points (candidalysin variant vs reference candidalysin). *C. dubliniensis* candidalysin variant C vs vehicle-treated cells = no significant difference. Statistical analysis was performed using a two-way ANOVA with Bonferroni *post hoc* multiple comparison test; *P < 0.05, **P < 0.01, ***P < 0.001, and ****P < 0.0001.

Candidalysins induce calcium influx into epithelial cells (7, 17). We therefore treated epithelial cells with 70 µM of each candidalysin variant for 40 min and quantified their ability to induce calcium influx (Fig. 3). The candidalysin from *C. albicans* SC5314, *C. dubliniensis* CD36, and *C. tropicalis* MYA-3404 were used as positive controls. The candidalysin from *C. albicans* SC5314 induced a gradual influx of calcium over time. While all variants of *C. albicans* candidalysin induced significant levels of calcium influx when compared to vehicle-treated epithelial cells, the majority (B, E, F, G, and H) induced calcium influx that was more rapid and elevated compared to SC5314 candidalysin (Fig. 3A). In contrast, variant C induced calcium influx similarly to SC5314 candidalysin, while variant I and J were observed to induce influx quicker, but to a lesser extent overall. Finally, variant D induced only modest calcium influx at 15 min when compared with SC5314 candidalysin. Calcium influx induced by *C. dubliniensis* candidalysin variant B was similar to but less potent than CD36 candidalysin (Fig. 3B), while variant C induced minimal influx. In contrast, *C. tropicalis* variant B induced calcium influx more rapidly and potently when compared to MYA-3404 candidalysin (Fig. 3C).

Taken together, these data indicate that naturally occurring candidalysin variants found in *C. albicans*, *C. dubliniensis*, and *C. tropicalis* differ in their ability to induce calcium influx.

### Candidalysin variants induce differential activation of MAPK signaling and cytokine secretion

**Fig 4:**
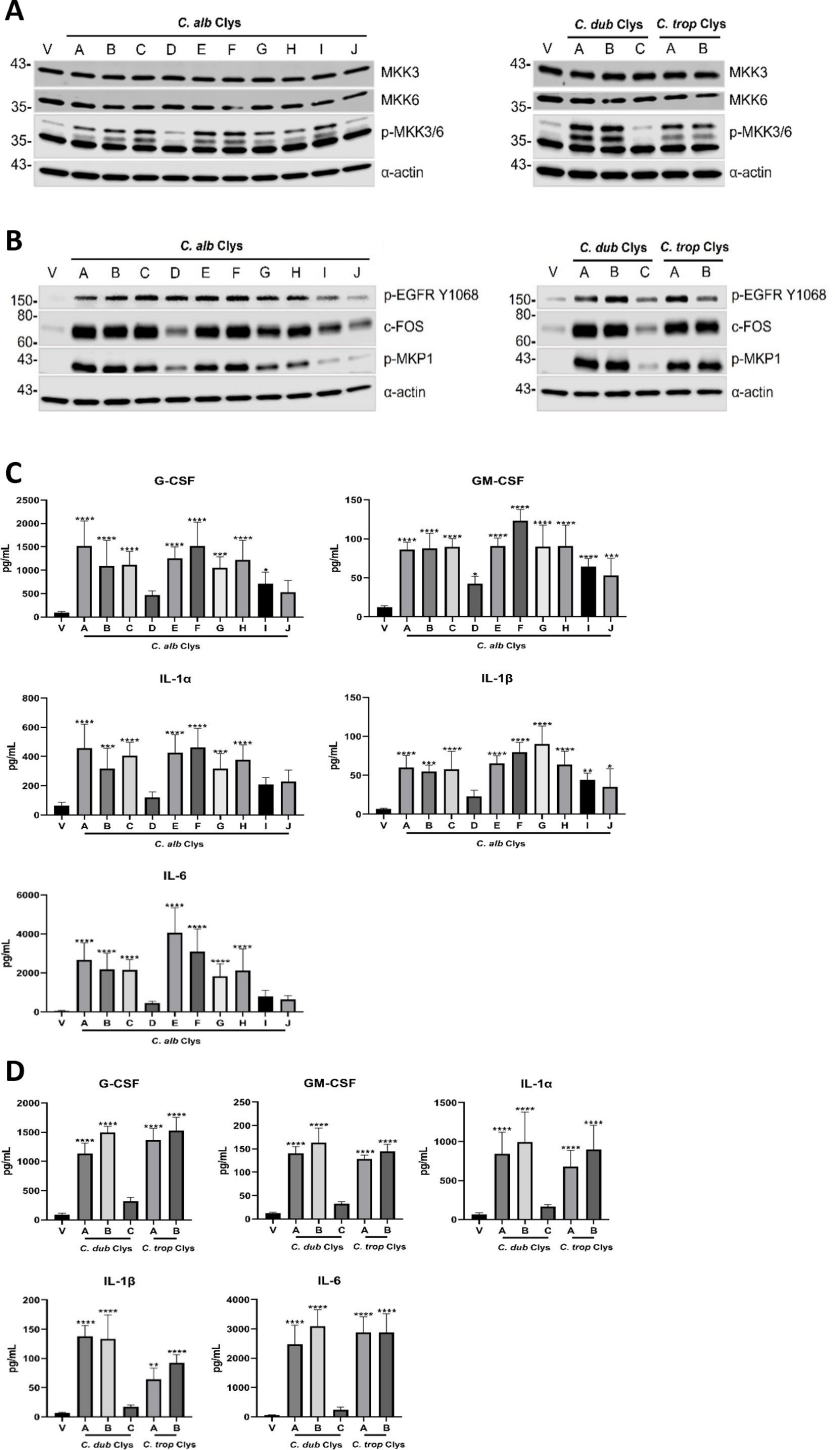
Candidalysin variants induce differential activation of MAPK signaling and cytokine secretion. Western blot analysis of TR146 epithelial cells treated with 15 µM of each candidalysin variant from *C. albicans*, *C. dubliniensis,* and *C. tropicalis* for (**A**) 30 min and (**B**) 2 h. Protein lysates (10 µg total protein) were probed with antibodies specific for MKK3, MKK6, and p-MKK3/6 (**A**), and p-EGFR, c-Fos, p-MKP1 (**B**). α-actin was used as a loading control. One representative blot presented from *n* = 3 biological repeats. Quantification of G-CSF, GM-CSF, IL-1α, IL-1β, and IL-6 secreted from TR146 epithelial cells treated with 15 µM of each candidalysin variant from (**C**) *C. albicans*, and (**D**) *C. dubliniensis* and *C. tropicalis* for 24 h. Data are the mean + SD of *n* = 3 biological repeats (*C. albicans* candidalysin variant A vs variant B; no significant difference for all cytokines tested). Statistical analysis was applied relative to vehicle-treated cells using a one-way ANOVA with a *post hoc* Bonferroni multiple comparison test; *P < 0.05, **P < 0.01, ***P < 0.001, and ****P < 0.0001.

Candidalysin from *C. albicans* SC5314 activates two distinct mitogen-activated protein kinase (MAPK) pathways in epithelial cells; the EGFR-ERK1/2-c-Fos axis, and the MKK3/6-p38 axis, which drive cytokine production (7, 15, 27). We therefore assessed the ability of each candidalysin variant to activate MAPK signaling. Early activation of the MKK3/6-p38 axis was investigated in epithelial cells treated with 15 µM of each candidalysin variant for 30 min (Fig. 4A). All candidalysin variants from *C. albicans* induced phosphorylation of MKK3 and MKK6 compared to vehicle-treated cells, except for *C. albicans* variant D and J (Fig. 4A). Candidalysins from *C. dubliniensis* CD36 *and C. tropicalis* MYA-3404 induced more MKK3/6 phosphorylation than *C. albicans* SC5314 candidalysin, with CD36 candidalysin being the most potent. All *C. dubliniensis* and *C. tropicalis* candidalysin variants except *C. dubliniensis* variant C induced MKK3/6 phosphorylation (Fig. 4A).

Activation of the EGFR-ERK1/2-c-Fos signaling axis was also investigated by assessing levels of epidermal growth factor receptor (EGFR) phosphorylation, MAPK phosphatase 1 (MKP1) phosphorylation, and c-Fos expression following treatment with 15 µM of each candidalysin variant for 2 h. The candidalysin of *C. albicans* SC5314, *C. dubliniensis* CD36, and *C. tropicalis* MYA-3404 robustly activated EGFR-ERK1/2-c-Fos signaling when compared to vehicle-treated cells (Fig. 4B). The majority of candidalysin variants induced a similar activation profile except for *C. albicans* variant D, I, and J, and *C. dubliniensis* variant C, which induced a diminished signaling response (Fig. 4B). Although *C. tropicalis* candidalysin variant B induced less EGFR phosphorylation when compared to variant A, levels of c-Fos expression and MKP1 phosphorylation were comparable (Fig. 4B). Densitometry analysis of the observed differences in epithelial signaling responses is presented in Fig. S7 (MKK3, MKK6, p-MKK3/6), and Fig. S8 (p-EGFR, c-Fos, p-MKP1).

Candidalysin-induced MAPK signaling drives cytokine secretion from epithelial cells (7, 15, 17, 27). To determine if the candidalysin variants induced similar responses, epithelial cells were treated with 15 and 70 µM of each candidalysin variant for 24 h and secretion of G-CSF, GM-CSF, IL-1α, IL-1β, and IL-6 was quantified (Fig. 4C and D; Fig. S9A and B). The majority of *C. albicans* candidalysin variants (15 µM) stimulated significant levels of cytokine secretion when compared to vehicle-treated cells except for variant D (G-CSF, IL-1α, IL-1β, and IL-6), variant I (IL-1α and IL-6), and variant J (G-CSF, IL-1α, and IL-6; Fig. 4C). However, the majority of these differences were abrogated with higher concentrations (70 µM) of each candidalysin (Fig. S9A).

While *C. dubliniensis* CD36 candidalysin and variant B (15 µM) stimulated similar levels of cytokine secretion, variant C was significantly reduced in its ability to stimulate secretion of all cytokines tested (Fig. 4D). In contrast, treatment of epithelial cells with 70 µM of *C. dubliniensis* CD36 candidalysin and variant B induced less cytokine secretion, most likely due to excess cytotoxicity, while variant C induced higher levels of cytokine secretion (Fig. S9B). No significant differences in cytokine secretion were observed between *C. tropicalis* MYA-3404 candidalysin and variant B at all concentrations tested (Fig. 4D; Fig. S9B).

Collectively, these data suggest that natural variants of candidalysin toxins from *C. albicans*, *C. dubliniensis*, and *C. tropicalis* vary in their ability to drive EGFR phosphorylation, MAPK signaling, and cytokine secretion.

## DISCUSSION

Damage caused to host cells and tissues during microbial infection frequently relies upon the production of toxins (28–30). Candidalysin is a secreted cytolysin that damages plasma membranes, mediates nutrient acquisition (31), promotes translocation (24), and activates innate immune responses during mucosal infection by *C. albicans* (7, 25, 26, 32–35). Recent investigations have revealed differences in candidalysin amino acid sequence in different strains of *C. albicans* (12, 18) and in related *Candida* species (17). Given these observations, we investigated the distribution of candidalysin sequence variation and identified new candidalysins in *C. albicans*, *C. dubliniensis*, and *C. tropicalis* (summarized in Table 2).

**TABLE 2:** Summary of biological and biophysical characteristics of candidalysin variants^*a*^

Candidalysin	Amphipathicity	α helicity	Cellular damage	Membrane permeabilization	Mitochondrial dysfunction	Calcium influx	MKK3/6 signaling	EGFR/MAPK signaling	Cytokine secretion
***C. alb* var A** SIIGIIMGILGNIPQVIQIIMSIVKAFKGNK	++++++	+++	+++	+++	+++++	+++	++	++++	+++
***C. alb* var B** SII**S**IIMGILGNIPQVIQIIMSIVKAFKGNK	++++++	++++	+++	nd	+++	++++++	++	++++	+++
***C. alb* var C** SIIGIIMGIL**T**NIPQVIQIIMSIVKAFKGNK	++++++	++++	++++	nd	+++++	+++	+++	++++	+++
***C. alb* var D** SIIGIIMGILGNIPQVIQIIMSIV**R**AFKGNK	++++++	++++	−	+	+	++	−	++	+
***C. alb* var E** SII**S**IIMG**L**LGNIPQVIQIIMSIVKAFKGNK	++++++	++++	++++	nd	++++	++++++	+++	++++	+++
***C. alb* var F** SII**S**IIMGIL**T**NIPQVIQIIMSIV**R**AFKGNK	++++++	+++	++++	nd	++++	++++	+++	++++	+++
***C. alb* var G** SII**S**IIMGIL**S**NIPQVIQIIMSIV**R**AFKGNK	++++++	+++++	++++	nd	++++	++++	++	+++	+++
***C. alb* var H** SIIGIIMG**L**L**T**NIPQVIQIIMSIV**R**AFKGNK	++++++	+++	+++	nd	+++	+++++	++	+++	+++
***C. alb* var I** SIIGIIMG**L**L**T**NIPQVI**N**IIMSI**I**KAFKGNK	++++++	++++	+	−	+++	+++	+++	++	++
***C. alb* var J** S**FLS**II**TAL**LGNIPQ**I**IQIIM**G**IVKAF**R**GNK	++++++	++++	+++	++	+++	+++	−	+	++
***C. dub* var A** SIIGILTAILNNVPQIINVITTIIKSITGNK	++++++	+++++	++++++	+++++	++++++	+++++	+++++	++++	+++
***C. dub* var B** SIIGILTAILNN**I**PQIINVITTIIKSITGNK	++++++	++++	++++	nd	++++++	++++	+++++	++++	+++
***C. dub* var C** SIIGILTAILNNVPQIINVI**M**TIIKSITGNK	++++++	+++++	−	−	+	+	−	+	+
***C. trop* var A** ISFAGIVSSIINQLPSIIQIIGNIIKAGLVK	+++++	++++	++++	nd	+++++	+++	+++	++++	+++
***C. trop* var B** **L**SFAGIV**G**SIINQLPSIIQIIGNIIKAGLVK	+++++	+++++	++++	nd	++++++	++++	+++	+++	+++

^*a*^ “++++++” = comparatively high activity, “+” = comparatively low activity, “−” = equivalent to vehicle, “nd” = not determined. Amino acid variations from the respective reference candidalysin are indicated (boldface and underlined).

Treatment of epithelial cells with individual candidalysin variants resulted in differing levels of cellular damage in a concentration-dependent manner. When applied in combination, candidalysin variants did not have an additive or synergistic effect on cellular damage. *C. albicans* candidalysin variant D caused significantly less damage compared to variant A at all concentrations tested but did not reduce the ability of variant A to cause damage, suggesting that the functionality of the more active variant is not impaired when candidalysin variants are present in combination.

The inability of *C. albicans* candidalysin variant D, I, and J and *C. dubliniensis* variant C to cause significant levels of cellular damage at low concentrations (15 µM) was unexpected and prompted a more detailed analysis using DPhPC planar bilayers to investigate the ability of each variant to interact with and permeabilize a membrane. Variant D displayed reversible membrane binding and transient membrane permeabilization but did not induce complete membrane rupture over the experimental time-course. In keeping with the nature of this transient membrane permeabilization, epithelial cells treated with 15 and 70 µM of *C. albicans* variant D exhibited only minimal damage, which may be explained by the induction of Alg-2/Alix/ESCRT-III-dependent blebbing which functions to repair membranes damaged by candidalysin (13).

*C. albicans* variant A and variant D candidalysin differ by a single arginine (K25R) located in the C-terminal region of the toxin, suggesting that this region may play important roles in membrane rupture. Variant I exhibited minimal bilayer interaction and failed to cause permeabilization, but damaged epithelial cells in a concentration-dependent manner, suggesting that there may be differences in how candidalysins interact with chemically homogeneous lipid bilayers and cellular plasma membranes. These observations further suggest that regions in the N- and C-terminus of the toxin may be critical for biological functionality. Finally, although delayed when compared with candidalysin variant A, variant J induced bilayer rupture but caused only modest levels of cellular damage at low concentrations (15 µM). Variant J has nine amino acid changes, with many located in the N-terminal region. These observations suggest that changes in the amino acid sequence of the N-terminal region are likely important for interaction with natural but not synthetic bilayers.

Mitochondrial activity is negatively affected in oral epithelial cells in response to candidalysin from *C. albicans* strain SC5314 as evidenced by decreased metabolic activity, depletion of intracellular adenosine triphosphate, and destabilization of mitochondrial membrane potential (14). Calcium influx is considered a key feature of the epithelial response to candidalysin (7, 25, 32, 35), and candidalysin activity was proposed to influence mitochondrial function in a calcium-dependent manner (14). However, while candidalysin variant C, E, F, and G induced similar or greater calcium influx than candidalysin variant A and also caused a reduction in metabolic activity, candidalysin variant B and H permitted more metabolic activity but induced greater calcium influx compared with variant A. Likewise, candidalysin variant B from *C. tropicalis* induced more calcium influx when compared to MYA-3403 variant A but also caused a marked reduction in metabolic activity. These data imply that the relationship between calcium influx and mitochondrial health is complex and that the effect of different candidalysins on mitochondrial function may not always be calcium-dependent.

Candidalysin from *C. albicans* strain SC5314 (variant A) induces rapid influx of calcium ions into epithelial cells (7, 17), while intracellular chelation of calcium with BAPTA-AM correlates with diminished MAPK signaling and cytokine secretion (35). In keeping with these observations, candidalysin variant D from *C. albicans* and variant C from *C. dubliniensis* both caused only modest levels of calcium influx, and reduced MAPK signaling and cytokine secretion (particularly IL-6). In contrast, candidalysin variant I and J from *C. albicans* also caused a reduction in MAPK signaling and cytokine secretion but induced rapid calcium influx (within 5 min) that did not exceed the level of influx caused by SC5314 candidalysin. This suggests that a threshold concentration of intracellular calcium may be required to induce host signaling responses.

Candidalysin-induced secretion of IL-6 from epithelial cells is driven through the MKK3/6-p38 signaling axis (15). As such, diminished IL-6 secretion correlated with an inability of some candidalysin variants (*C. albicans* variants D and J; *C. dubliniensis* variant C) to activate the MKK3/6-p38 signaling pathway. However, additional epithelial responses were also reduced (e.g., c-Fos, G-CSF, and GM-CSF), which are induced through the EGFR-ERK1/2-c-Fos pathway (15). This suggests that differences in the candidalysin amino sequence can lead to a general impairment in epithelial activation rather than a specific inability to activate the MKK3/6-p38 signaling axis.

A previous analysis of 78 *C*. *albicans* vaginal isolates revealed 31 that contained a candidalysin variant with the same amino acid sequence as strain SC5314 (referred to as variant A; this study), 45 with a candidalysin variant that was first observed in strain 529L (referred to as variant J; this study), and two candidalysin variants in which the glycine at position four was replaced with arginine (G4R: SII**R**IIMGILGNIPQVIQIIMSIVKAFKGNK) (12). Although we did not identify the G4R variant in our analysis, manual mutation of the glycine at position four to tryptophan (G4W: SII**W**IIMGILGNIPQVIQIIMSIVKAFKGNK) caused a reduction in epithelial c-Fos expression and MKP1 phosphorylation compared with SC5314 candidalysin (variant A), suggesting that the glycine at position 4 may be important for toxin function (36). Our analysis identified a naturally occurring candidalysin variant with a serine at position four (variant B: SII**S**IIMGILGNIPQVIQIIMSIVKAFKGNK). Except for mitochondrial and calcium responses, no significant differences were observed in the level of cellular damage, MAPK signaling, or cytokine secretion when variant B was compared with variant A. Thus, while a mutated candidalysin harbouring a severe (G4W) amino acid substitution induces a diminished epithelial MAPK signaling response, a naturally occurring and less severe substitution (G4S) has only modest biological impact. This suggests that the glycine at position 4 may not be critical for toxin activity, and differences in epithelial signaling responses may be influenced by steric bulk.

A recent investigation (18) of epithelial phenotypes associated with vaginal colonization and vulvovaginal candidiasis (VVC) highlighted similar variation in the sequence of *C. albicans* candidalysin as identified in this study with the prevalence F(8/22) > A(7/22) > G(2/22) = H(2/22) > J(1/22), plus two additional variants (SII**S**IIMGIL**T**NIPQVIQIIMSIV**R**AF**R**GNK, and SIIGIIMGILGNIPQV**V**QI**T**MSIVKAFKGNK). Isolates associated with vaginal colonization contained either candidalysin variant A(5/22) or variant F(3/22), whereas those associated with VVC contained candidalysin variants F(5/22) > A(2/22) = G(2/22) = H(2/22) > J(1/22) (18). Our analysis demonstrates that candidalysin variants found in isolates associated with vaginal colonization (e.g., variant F) are highly active, while variants associated with VVC (e.g., variant J) exhibit reduced activity when compared to variant F. However, candidalysin variant J caused levels of damage that were similar to variant A when applied to vaginal epithelial cells (12), and oral epithelial cells (this study). This apparent discordance (a more active candidalysin variant in a *C. albicans* isolate associated with vaginal colonization rather than VVC) may be explained by differences in candidalysin secretion. Indeed, secretome analysis demonstrated that only isolates associated with VVC secreted detectable levels of their respective candidalysin *in vitro* (18).

Our biological and biophysical data combined with published sequence analysis (12, 18) support the hypothesis that variation in candidalysin sequence is widespread in isolates of *C. albicans*, but less so in isolates of *C. dubliniensis* and *C. tropicalis*. Fewer candidalysin variants were found in *C. tropicalis* compared to *C. albicans* (frequency of 0.026 and 0.054, respectively). However, an accurate comparison with *C. dubliniensis* is precluded by the small number of isolates available for analysis.

Our experimental findings reveal differences in the activity of candidalysin variants identified in these species, raising the questions; how and why did such natural variation occur? One potential explanation may be the selective pressures imposed on *Candida* species during their co-evolution with the host. It is possible that some candidalysin variants may confer improved fungal commensalism, pathogenicity, or persistence in the host. However, care must be taken when interpreting toxin activity *in vitro* in the context of fungal infection *in vivo*. “Optimal” fungal pathogenicity, particularly at mucosal surfaces, is dependent upon numerous criteria including the ability to grow and maintain robustly adherent hyphae, the formation of an invasion pocket, high levels of *ECE1* gene expression, appropriate intracellular processing of Ece1p to yield mature candidalysin (7, 11, 12), and the accumulation of secreted toxin within the invasion pocket (8, 13) at concentrations sufficient to cause pathology. The efficiency of Ece1p processing by kexin proteinases in *C. albicans* strain SC5314 (candidalysin variant A) and 529L (candidalysin variant J) is influenced by the amino acid sequence between the P2 and 3 junctions of Ece1p (9). Indeed, while the current study focusses on cellular and biophysical responses to candidalysin variant peptides *in vitro*, our analysis also revealed differences in amino acid sequence between Ece1p P2 and P3 (TAIT (SC5314-like) in 312 alleles, TDLA (529L-like) in 37 alleles, TDIA in 11 alleles, and TALA in 4 alleles), highlighting that differences in Ece1p processing efficiency are likely to influence isolate pathogenicity. Further virulence attributes and activities are known to be critical for candidalysin secretion, delivery, and host cell damage (8). While candidalysin is crucial for the full ability to damage host cells, additional isolate-specific differences in multiple aspects of virulence likely influence overall virulence, which itself is also influenced by tissue type, composition of the surrounding microbiota, and host genetics. Accordingly, while the activity of candidalysin variants may vary *in vitro*, these differences may not correlate directly with the pathogenicity of the associated fungal isolate *in vivo* (37).

In summary, this study identifies variations in candidalysin amino acid sequence and biological activity within populations of *C. albicans*, *C. dubliniensis*, and *C. tropicalis*, which may have profound implications in the wider context of mucosal infection and immunopathology.

## MATERIALS AND METHODS

### Identification of candidalysin variants in *C. albicans*

Single-nucleotide polymorphisms in the *ECE1* gene of 182 genome-sequenced *C. albicans* isolates (19) were retrieved and used as input in PHASE v2.1.1 (21, 38). Forty-seven *C. albicans ECE1* haplotypes were identified, corresponding to 43 *C*. *albicans* Ece1 variants (data not shown) and 10 *C*. *albicans* candidalysin variants, noted A–J. For each isolate, the haplotype composition and hence candidalysin variant composition was retrieved from the PHASE analysis output and used to build a summary table (Fig. S1).

### Identification of candidalysin variants in *C. dubliniensis*

*C. dubliniensis* candidalysin variants were identified using whole-genome sequencing data from 10 isolates (Table S1). These isolates were recovered from either human or avian-associated sources and disparate geographical locations and represented each of three previously identified MLST clades and four previously identified ITS genotypes. One of the 10 isolates (AV5) was recovered from avian-associated excrement (39) and another isolate (Is30) was included as it showed enhanced virulence in a murine model in comparison with other *C. dubliniensis* isolates (40). The Wü284 isolate was also included in the current study as this strain is widely used in *C. dubliniensis* cloning experiments (41). The previously sequenced reference CD36 type strain (42) was also included in the 10 isolates in order to serve as a comparative control for sequence and data analysis (Fig. S2).

Genomic DNA was extracted, and genomes were sequenced at the Biomics Pole—Genomic Platform of Institut Pasteur using Illumina sequencing technology. Single reads of 50 bp were obtained. Reads have been deposited at the NCBI Sequence Read Archive under BioProject ID: PRJNA1048350. Reads were mapped against the *C. dubliniensis* strain CD36 reference genome downloaded from the *Candida* Genome Database version s01-m02-r30 (43) using the Burrows–Wheeler Alignment tool, BWA version 0.7.755, with the BWA-MEM algorithm. SAMtools version 1.256 and Picard tools version 1.94 (http://broadinstitute.github.io/picard) were then used to filter, sort, and convert SAM files.

SNPs were called using Genome Analysis Toolkit version 3.6 (44) according to the GATK Best Practices. SNPs and indels were filtered using these following parameters: VariantFiltration, QD < 2.0, LowQD, ReadPosRankSum < −8.0, LowRankSum, FS > 60.0, HightFS, MQRankSum < −12.5, MQRankSum, MQ < 40.0, LowMQ, HaplotypeScore > 13.0, HaploScore. Coverages were also calculated using the Genome Analysis Toolkit.

### Identification of candidalysin variants in *C. tropicalis*

TrimGalore v. 0.4.3 (45) was used to remove adapters from 78 *C*. *tropicalis* genomes (20) and to trim reads to minimum mean qualities of 30 and minimum lengths of 35. Trimmed reads were aligned to the *C. tropicalis* reference sequence MYA-3404 using BWA-MEM v.0.7.11 (46). BAM files were sorted and indexed, and duplicate reads were marked using Genome Analysis Toolkit version 3.7 (GATK) (44). Variants were called using GATK HaplotypeCaller in “--genotyping_mode DISCOVERY.” Variants were filtered for minimum genotype qualities of 20 and minimum read depths of 10. SNPs were extracted using GATK SelectVariants (44). Variants in the *ECE1* ortholog (CTRG_00476) were manually analyzed using the Integrative Genomics Viewer, IGV (47). Variants at 30 amino acids were identified. However, most lay outside the candidalysin peptide sequence. Only two candidalysin alleles were identified, with phasing evident from the BAM alignments. One allele is present in all sequenced *C. tropicalis* isolates, and the second is also present in six hybrid isolates that share only one parent with the other isolates (Fig. S3).

### Candidalysin peptides

The amino acid sequence of candidalysin peptides used in this study are listed in Table 1. All peptides were purchased from Peptide Protein Research Ltd (UK). Each peptide was synthesized using standard Fmoc chemistry and purified by HPLC to a minimum purity of 95%. Peptide purity and experimental molecular mass were further verified by LC-MS/MS. Each peptide was reconstituted in sterile water to a stock concentration of 3.5 mM. Stocks were aliquoted and stored at −20°C.

### Circular dichroism spectroscopy

Circular dichroism was performed using a Chirascan Plus CD spectrometer (Applied Photophysics) at 20°C. All candidalysin peptides were diluted to 70 µM in buffer (10 mM Tris pH 7.0, 50 mM NaCl) and acquired using a 0.5 mm path length cuvette. CD was measured at wavelengths 190 nm to 260 nm, with 1 nm bandwidth, reads every 1.5 s, and 3 repeats for each acquired point. Acquired CD spectra were averaged, adjusted for background buffer levels, and smoothed using GraphPad Prism 9.

### Mammalian cell culture

Experiments were performed using TR146 human oral epithelial cells (48) obtained from the European Collection of Authenticated Cell Cultures (ECACC). Cells were routinely tested for mycoplasma using PCR. Cells were grown in Dulbecco’s modified Eagle’s medium (DMEM) F12 nutrient mixture, supplemented with 15% foetal bovine serum (FBS) and 1% penicillin-streptomycin at 37°C with 5% CO_2_.

### Treatment of epithelial cells with candidalysin variants

Prior to treatment, TR146 cells were cultured for 24 h until confluent and serum starved overnight. Cells were then replenished with serum free DMEM-F12 medium and treated with candidalysin variants for 24 h (cellular damage), 6 h (mitochondrial fitness), 40 min (calcium influx), 30 min and 2 h (MAPK signaling), and 24 h (cytokine secretion). The concentration of candidalysins used during experimentation (15 µM and 70 µM) was based on historical observations of *C. albicans* candidalysin function (7), in which *in vitro* analysis demonstrated 70 µM to be lytic, and 15 µM to be borderline-lytic, and the observation that *C. dubliniensis* and *C. tropicalis* candidalysins are more potent than the *C. albicans* candidalysin when applied to epithelial cells at equivalent concentrations (17). For cellular damage assays (LDH), candidalysin concentrations of 15 µM and 70 µM were included in the analysis to maximize observable differences in candidalysin potency at the lower concentration (15 µM). For metabolic activity assays (MTT), 15 µM candidalysin was included to rule out any effects that might arise from overt toxicity. For calcium influx assays, 70 µM was used to compare calcium influx profiles. For analysis of epithelial signal transduction (western blotting), 15 µM candidalysin was used as the protein yield from epithelial cells treated with 70 µM was often very low due to excessive cellular damage.

### Quantification of cellular damage

Exhausted culture medium was collected and assayed for lactate dehydrogenase (LDH) activity using a Cytox 96 Non-Radioactive assay kit (Promega) according to the manufacturer’s instruction. Recombinant porcine L-lactate dehydrogenase (Sigma) was used to create a standard curve.

### Quantification of bilayer permeabilization

Horizontal lipid bilayer experiments were performed using an Orbit e16 platform (Nanion). Lipid bilayers were prepared using 1,2-diphytanoyl-*sn*-glycero-3-phosphocholine (DPhPC) lipids dissolved in octane (25 mg/mL) and formed over a 16 channel multielectrode cavity array chip (Ionera). A buffer containing 0.1 M KCl and 20 mM HEPES (pH 7.4) was added into the cavities above and below the bilayers and a constant voltage of −50 mV was applied. Candidalysin peptides were dissolved in water and a final concentration of 10 µM was added to the bilayers. Current changes were monitored for 40 min at room temperature. Data analysis was performed using Clampfit software v10.3 (Molecular Devices).

### Mitochondrial activity assay

3-(4,5-dimethylthiazol-2-yl)−2,5-diphenyltetrazolium bromide (MTT; Sigma) was prepared to 0.5 mg/mL in serum free medium and sterilized through a 0.2 µM filter. Cells were treated with each candidalysin variant (in triplicate per assay) for 6 h. Cells were replenished with 100 µL of MTT solution and incubated for 3 h at 37°C, 5% CO_2_. This was followed by the addition of 150 µL of solubilization solution [37% hydrochloric acid (HCl) in 10% sodium dodecyl sulfate (SDS)] and incubated overnight at 37°C. Absorbance was measured at 620 nm wavelength using an Infinite F50 plate reader (TECAN). Cell viability was calculated as a percentage of untreated cells.

### Quantification of calcium influx

Epithelial cells were seeded into 96 well plates (Greiner), cultured for 24 h, and serum starved overnight. Cells were treated with a solution containing 2.5 µM Fura-2 AM and 500 µM probenecid in saline solution (NaCl (140 mM), KCl (5 mM), CaCl_2_ (2 mM), MgCl_2_ (1 mM), glucose (10 mM), and HEPES (10 mM) at pH 7.4) for 1 h at 37°C and 5% CO_2_. Following incubation, the solution was removed and replaced with a saline solution containing calcium. Candidalysin variants were added to each well, and readings were taken every 15 s for 40 min. Samples were excited at 340 and 380 nm, and fluorescence was detected at 520 nm using a FlexStation 3 microplate reader (Molecular Devices). Results were presented as a ratio between 340/380 nm and normalized with baseline fluorescence levels.

### Protein extraction

TR146 cells were lysed using a modified RIPA buffer (50 mM Tris-HCl pH 7.4, 150 mM NaCl, 1 mM EDTA, 1% Triton X-100, 1% sodium deoxycholate, 0.1% SDS) supplemented with 1% phosphatase (Sigma) and 1% protease inhibitors (Sigma-Aldrich). Lysates were incubated on ice for 30 min and centrifuged at 13,300 × *g* for 10 min at 4°C. Protein lysates were collected, and total protein concentration quantified using a bicinchoninic acid assay (Thermo Scientific).

### SDS-PAGE and western blotting

Total protein (10 µg) was separated by electrophoresis on 12% SDS-PAGE gels. Proteins were transferred onto a nitrocellulose membrane (GE Healthcare) and then blocked in 1× Tris-buffered saline (TBS) containing 0.1% Tween 20 and 5% fat-free milk powder. Membranes were then incubated with primary antibodies (1:1000) MKK3, MKK6, p-MKK3/6, p-EGFR Y1068, c-Fos, p-MKP1, or (1:10,000) α-actin overnight at 4°C, followed by secondary antibodies (1:10,000) for 1 h at RT, then exposed to Immobilon Western Chemiluminescent HFP substrate (Sigma-Aldrich) for 4 min. Images were acquired using a LI-COR imaging platform (Odyssey Fc, LI-COR). Human α-actin was used as a loading control.

### Densitometry analysis

Quantification of band intensity was performed using Image Studio Lite software. Band intensities for each lane were normalized to α-actin and data is displayed relative to the reference candidalysin (variant A)-treated cells.

### Antibodies

Primary antibodies specific for MKK3 (#8535), MKK6 (#8550), p-MKK3/6 (MKK3-Ser189 and MKK6-Ser207; #12280), p-EGFR Y1068 (#3777S), c-Fos (#2250S), and p-MKP1 (#2857S) were purchased from Cell Signaling Technology. α-Actin (#MAB1501) was purchased from Millipore. Secondary antibodies Peroxidase AffiniPure goat anti-rabbit (#111–035-144) and anti-mouse (#115–035-062) were purchased from Jackson ImmunoResearch Ltd.

### Quantification of cytokine secretion

Exhausted culture medium was collected, and cytokine secretion was quantified using a magnetic Fluorokine performance MAP cytokine multiplex kit (Bio-techne) and Bio-Plex 200 system (BioRad) according to the manufacturer’s instructions. Analyte concentrations for G-CSF, GM-CSF, IL-1α, IL-1β, and IL-6 were determined using Bioplex Manager 6.1 software.

### Statistical analysis of data

Data were tested for normality using a Shapiro-Wilk normality test. Normally distributed data were analyzed by one-way analysis of variance (ANOVA) with a *post hoc* Bonferroni comparison test to compare data sets from a minimum of three biological repeats using GraphPad Prism 9 software. A two-way ANOVA with a *post hoc* Bonferroni comparison test was used to compare calcium influx data (Fig. 3). A *P*-value of <0.05 was considered significant.

## Data Availability

Sequencing data relating to the *C. dubliniensis* isolates used in the study has been deposited at the NCBI Sequence Read Archive under BioProject ID: PRJNA1048350.

## SUPPLEMENTAL MATERIAL

Supplemental Material mbio.03351-23-s0001.pdf Supplemental figures and table.

ASM does not own the copyrights to Supplemental Material that may be linked to, or accessed through, an article. The authors have granted ASM a non-exclusive, world-wide license to publish the Supplemental Material files. Please contact the corresponding author directly for reuse.
