# Supplementary material for: Variations in candidalysin amino acid sequence influence toxicity and host responses
Source: mBio. 2024 Jul 2;15(8):e03351-23. doi: 10.1128/mbio.03351-23 (PMC11323794; doi:10.1128/mbio.03351-23)
Supplement: Supplemental Material — Supplemental figures and table. [file mbio.03351-23-s0001.pdf]

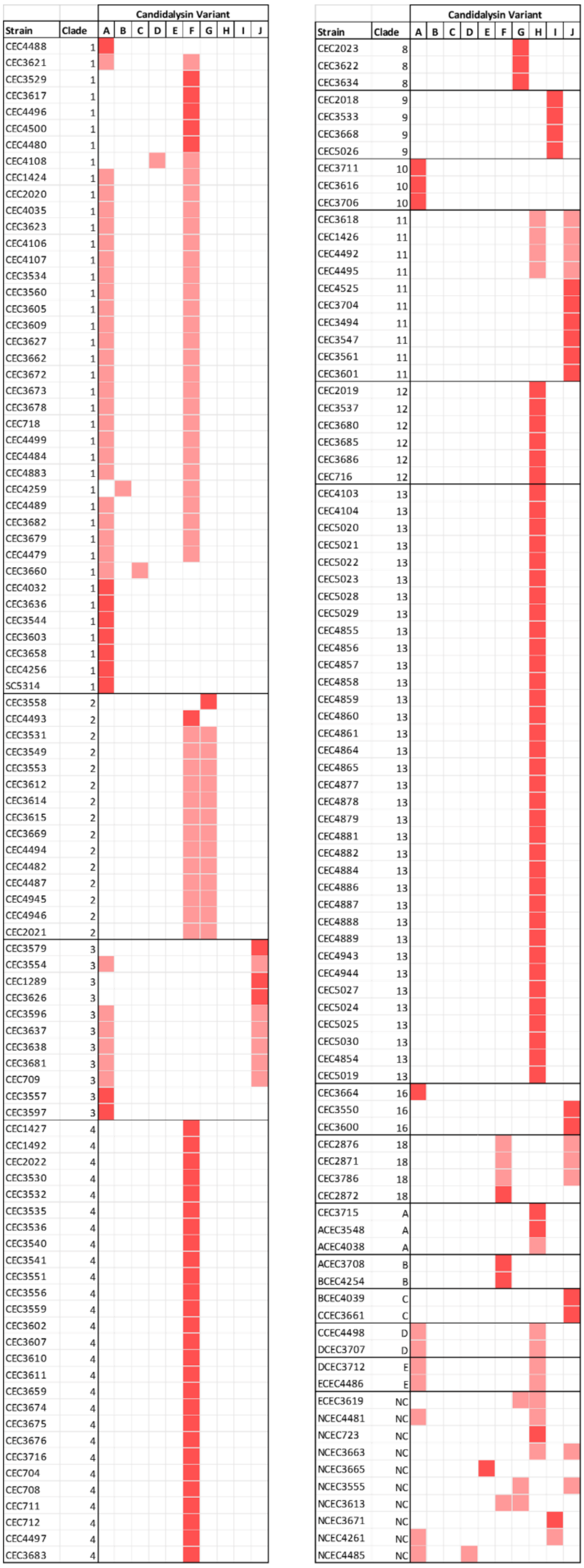

Candidalysin Variant	Amino acid sequence
C. alb Clys – Variant A	SIIGIIMGILGNIPQVIQIIMSIVKAFKGNK
C. alb Clys – Variant B	SIISIIMGILGNIPQVIQIIMSIVKAFKGNK
C. alb Clys – Variant C	SIIGIIMGILTNIPQVIQIIMSIVKAFKGNK
C. alb Clys – Variant D	SIIGIIMGILGNIPQVIQIIMSIVRAFKGNK
C. alb Clys – Variant E	SIISIIMGLLGNIPQVIQIIMSIVKAFKGNK
C. alb Clys – Variant F	SIISIIMGILTNIPQVIQIIMSIVRAFKGNK
C. alb Clys – Variant G	SIISIIMGILSNIPQVIQIIMSIVRAFKGNK
C. alb Clys – Variant H	SIIGIIMGLLTNIPQVIQIIMSIVRAFKGNK
C. alb Clys – Variant I	SIIGIIMGLLTNIPQVINIIMSIIKAFKGNK
C. alb Clys – Variant J	SFLSIITALLGNIPQIIQIIMGIVKAFRGNK

Supplemental Figure S1: Candidalysin variants observed in isolates of *C. albicans*. Single nucleotide polymorphisms of *C. albicans ECE1* were retrieved for 182 genome-sequenced isolates[19]. Haplotypes were determined using PHASE and the amino acid sequence of

candidalysin was inferred for each haplotype. For the 10 candidalysin variants (A to J) identified, the amino acid sequence is shown and amino acids that differ from those in the variant from strain SC5314 (variant A) are shown in red. For each isolate, the identified variants are shown; red, two copies of the same variant are present; pink, two different variants are present. Isolates have been ordered according to the cluster they belong (1-18 and A-E[19]; NC corresponds to isolates that cannot be associated to a genetic cluster).

		Candidalysin Variant		
Strain	Clade	A	B	C
CD36	1	■		
Wü284	1	■		
AV5	1	■		
Is30	1	■		
CD06037	2		■	
Can4	2		■	
CD514	3	■		
p7276	3			■
Eg207	3			■
p7718	3	■		

**Candidalysin Variant**	**Amino acid sequence**
C. dub Clys – Variant A	SIIGILTAILNNVPQIINVITTIIKSITGNK
C. dub Clys – Variant B	SIIGILTAILNNIPQIINVITTIIKSITGNK
C. dub Clys – Variant C	SIIGILTAILNNVPQIINVIMTIIKSITGNK

Supplemental Figure S2: Candidalysin variants observed in isolates of *C. dubliniensis*. Single nucleotide polymorphisms (SNPs) of *C. dubliniensis ECE1* were retrieved using whole genome sequencing data from 10 genome-sequenced isolates **(Supplemental Table S1)**. SNPs were called using Genome Analysis Toolkit version 3.6 according to the GATK Best Practices. For the three candidalysin variants (A to C) identified, the amino acids that differ from those in the variant from strain CD36 (variant A) are shown in red. All isolates were homozygous for a candidalysin variant.

		Candidalysin Variant	
Strain	Clade	A	B
ct01	A		
ct02	A		
ct03	A		
ct04	A		
ct05	A		
ct06	A		
ct07	A		
ct08	A		
ct09	A		
ct10	A		
ct11	A		
ct12	A		
ct13	A		
ct14	A		
ct15	A		
ct16	A		
ct17	A		
ct18	A		
ct19	A		
ct20	A		
ct21	A		
ct22	A		
ct23	A		
ct24	A		
ct26	A		
ct27	A		
ct28	A		
ct29	A		
ct30	A		
ct31	A		
ct32	A		
ct33	A		
ct34	A		
ct35	A		
ct36	A		
ct37	A		
ct38	A		
ct39	A		
ct40	A		
ct41	A		
ct43	A		
ct44	A		
ct45	A		
ct46	A		
ct47	A		
ct48	A		
ct49	A		
ct50	A		
ct51	A		
ct52	A		
ct53	A		
ct54	A		
ct55	A		
ct56	A		
ct57	A		
ct58	A		
ct59	A		
ct60	A		
ct61	A		
ct62	A		
ct63	A		
ct64	A		
ct65	A		
ct66	A		
ct67	A		
ct68	A		
ct69	A		
ct70	A		
ct71	A		
ct72	A		
ct73	A		
ct74	A		
ct25	B		
ct42	B		
ct75	B		
ct76	B		
ct77	B		
ct78	B		

Candidalysin Variant	Amino add sequence
C. trop Clys – Variant A	ISFAGIVSSIINQLPSIIQIIGNIIKAGLVK
C. trop Clys – Variant B	LSFAGIVGSIINQLPSIIQIIGNIIKAGLVK

Supplemental Figure S3: Candidalysin variants observed in isolates of *C. tropicalis*. Single nucleotide polymorphisms within the candidalysin region of the *ECE1* ortholog CTRG_00476

were retrieved from 78 Candida tropicalis isolates[20] and were manually analysed to identify changes in amino acid sequence. For each isolate, the corresponding amino acid sequence is coloured: red for two copies of one version; and pink for one copy of each version. Isolates are ordered by clade. The six isolates with two candidalysin peptides are hybrids that share only one parent with all other *C. tropicalis* isolates[20].

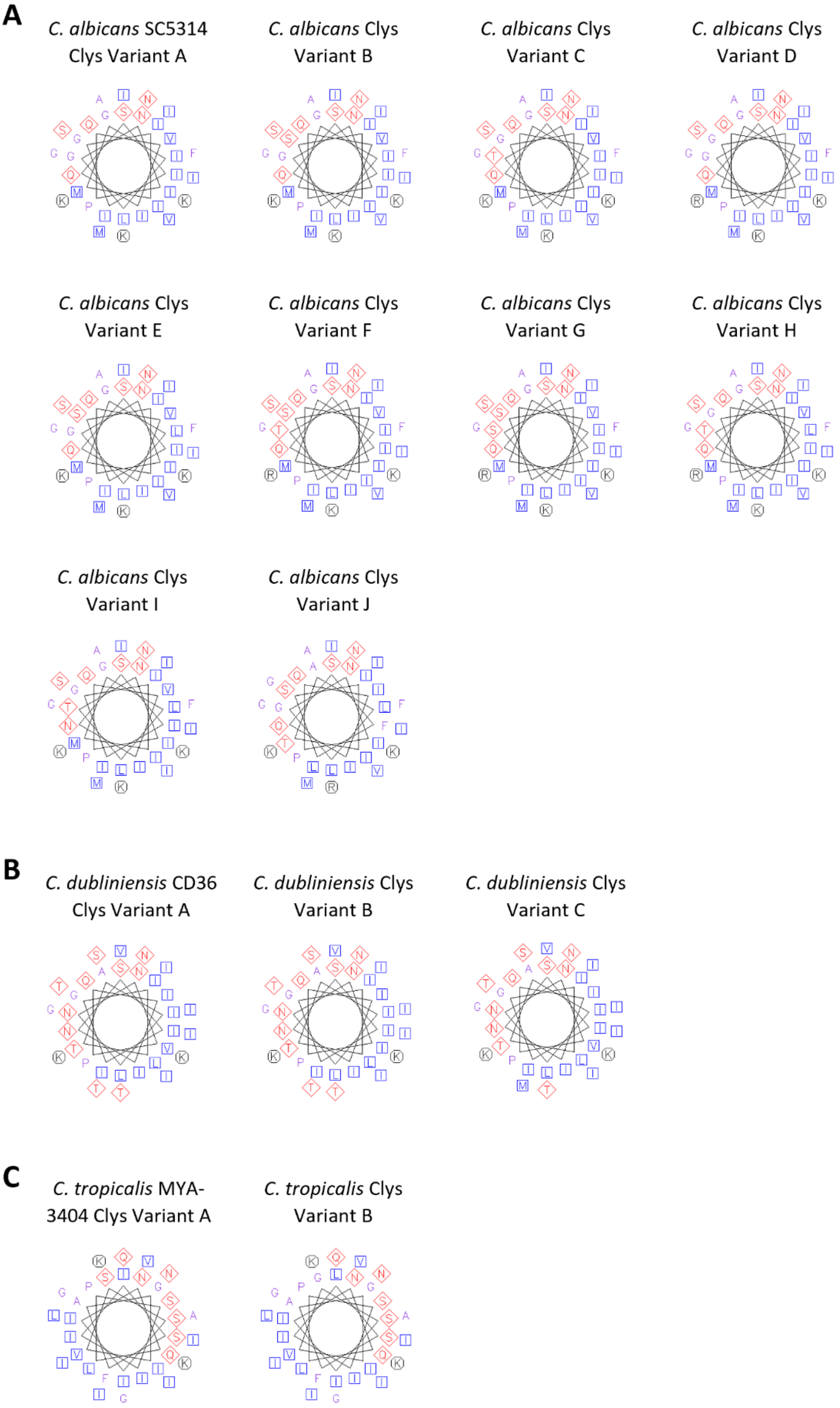

Supplemental Figure S4: Helical-wheel renderings of candidalysin variants from **(A)** *C. albicans*, **(B)** *C dubliniensis*, and **(C)** *C. tropicalis*. Hydrophobic amino acid residues are highlighted in blue.

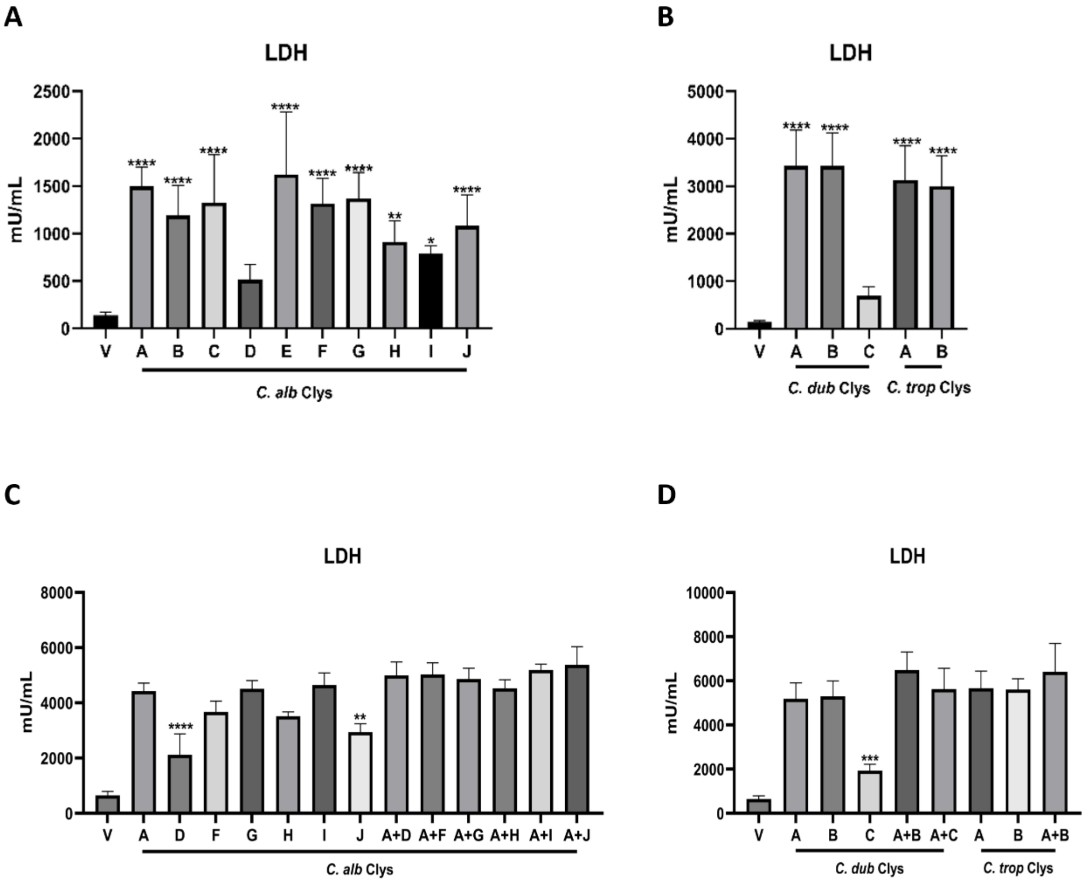

Supplemental Figure S5: Quantification of candidalysin-induced epithelial damage. TR146 oral epithelial cells were treated with 70 µM of each candidalysin variant from **(A)** *C. albicans*, and **(B)** *C. dubliniensis* and *C. tropicalis* for 24 h, and exhausted culture medium was quantified for LDH activity. Data are the mean + SD of n = 3 biological repeats. Statistical analysis was applied relative to vehicle-treated cells. *C. albicans* candidalysin variant A vs; variant D, P = 0.0097 (**); variant I, P = 0.0934 (not significant); and variant H, P = 0.2245 (not significant). *C. dubliniensis* variant A vs variant C, P = 0.0004 (***). Statistical analysis was performed using a one-way ANOVA with Bonferroni post hoc multiple comparison test; * P < 0.05, ** P < 0.01, *** P < 0.001, and P **** < 0.0001. TR146 oral epithelial cells were treated with 70 µM or a combination of 70 µM of each candidalysin variant from **(C)** *C. albicans*, and **(D)** *C. dubliniensis* and *C. tropicalis* for 24 h, and exhausted culture medium was quantified for LDH activity. Data are the mean + SD of n = 3 biological repeats. Statistical analysis was applied relative to the reference candidalysin (variant A)-treated cells using a one-way ANOVA with a post hoc Bonferroni multiple comparison test; ** P < 0.01, *** P < 0.001, and P **** < 0.0001

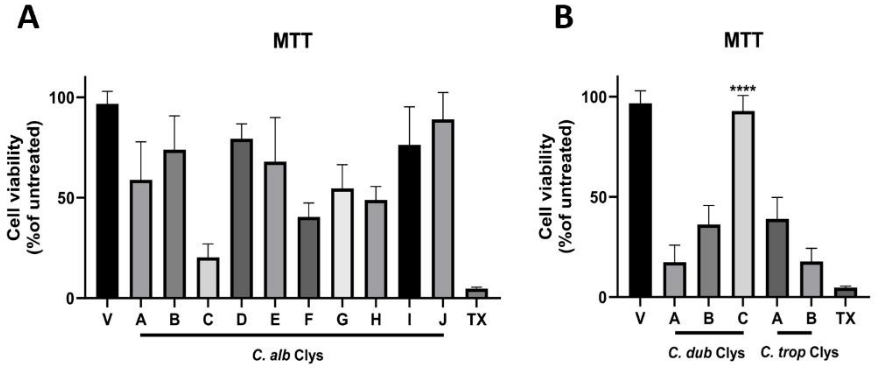

Supplemental Figure S6: Candidalysin variants induce changes in the metabolic activity of epithelial cells. TR146 epithelial cells were treated with 15 µM of each candidalysin variant from **(A)** *C. albicans*, and **(B)** *C. dubliniensis* and *C. tropicalis* for 6 h, and metabolic activity was quantified by MTT assay. Data is relative to untreated cells and presented as the mean + SD of n = 3 biological repeats. Statistical analysis was applied relative to the reference candidalysin (variant A)-treated cells. Statistical analysis was performed using a one-way ANOVA with a post hoc Bonferroni multiple comparison test; P **** < 0.0001.

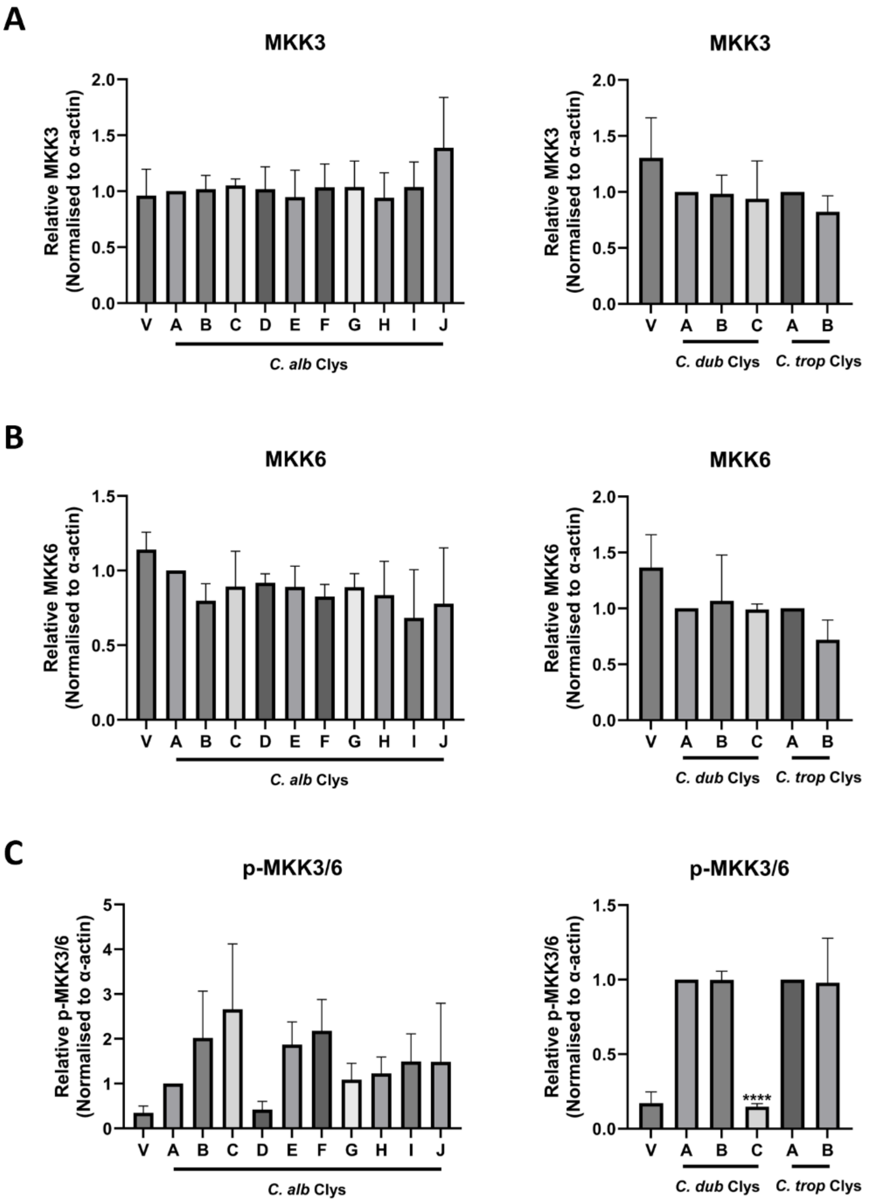

Supplemental Figure S7: Densitometry analysis of MKK3, MKK6 and p-MKK3/6 in TR146 oral epithelial cells following treatment with candidalysin variants. TR146 oral epithelial cells were treated with 15 µM of each candidalysin variant from *C. albicans*, *C. dubliniensis* and *C. tropicalis* for 30 min. Protein lysates (10 µg total protein) were probed with antibodies specific for MKK3, MKK6, and p-MKK3/6. Data represents mean + SD of band intensities normalised from n = 3 biological repeats. Data is relative to the reference candidalysin (variant A)-treated cells. Statistical analysis was applied relative to variant A-treated cells using a one-way ANOVA with a post hoc Bonferroni multiple comparison test; P **** < 0.0001.

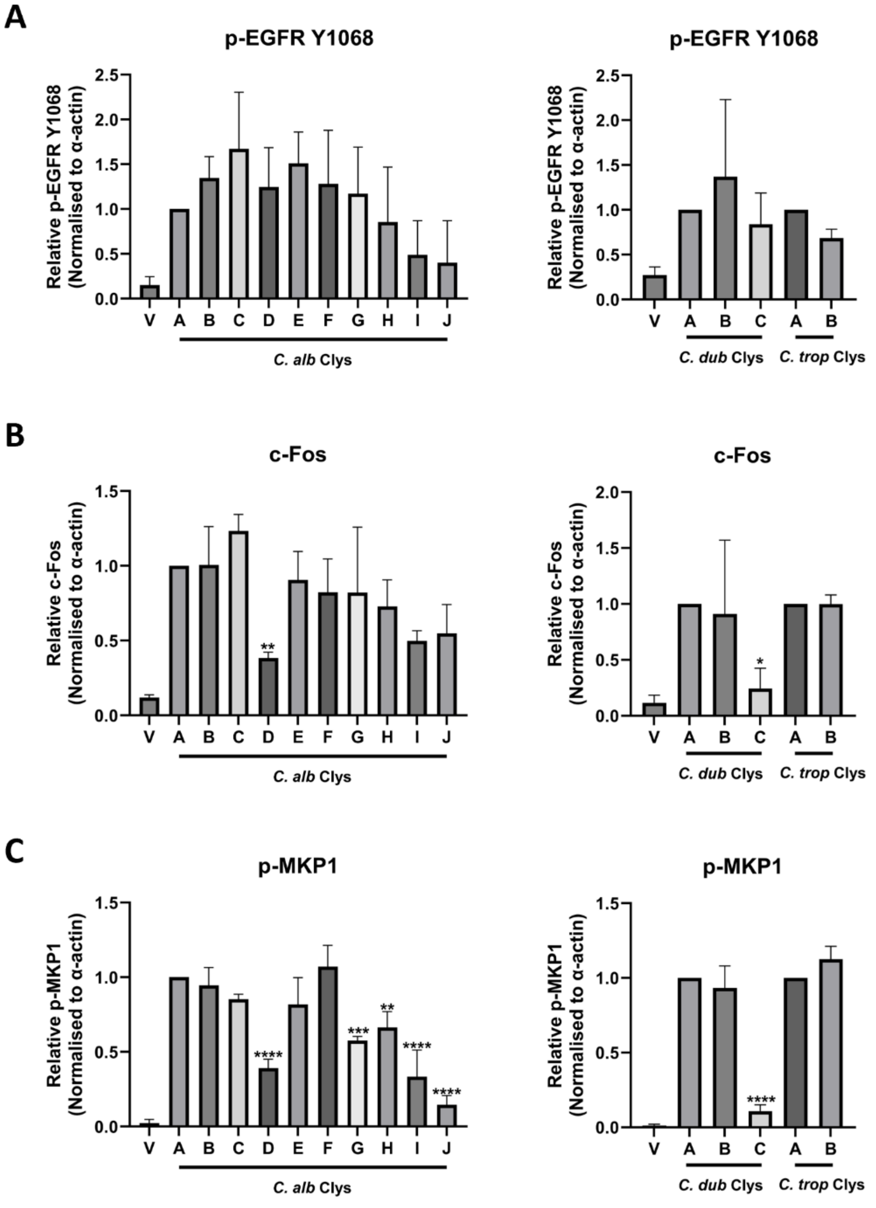

Supplemental Figure S8: Densitometry analysis of p-EGFR, c-Fos and p-MKP1 in TR146 oral epithelial cells following treatment with candidalysin variants. TR146 oral epithelial cells were treated with 15 µM of each candidalysin variant from *C. albicans*, *C. dubliniensis* and *C. tropicalis* for 2 h. Protein lysates (10 µg total protein) were probed with antibodies specific for p-EGFR Y1068, c-Fos and p-MKP1. Data represents mean + SD of band intensities normalised from n = 3 biological repeats. Data is relative to the reference candidalysin (variant A)-treated cells. Statistical analysis was applied relative to variant A-treated cells using a one-way ANOVA with a post hoc Bonferroni multiple comparison test; * $P < 0.05$, ** $P < 0.01$, *** $P < 0.001$, and P **** < 0.0001.

A

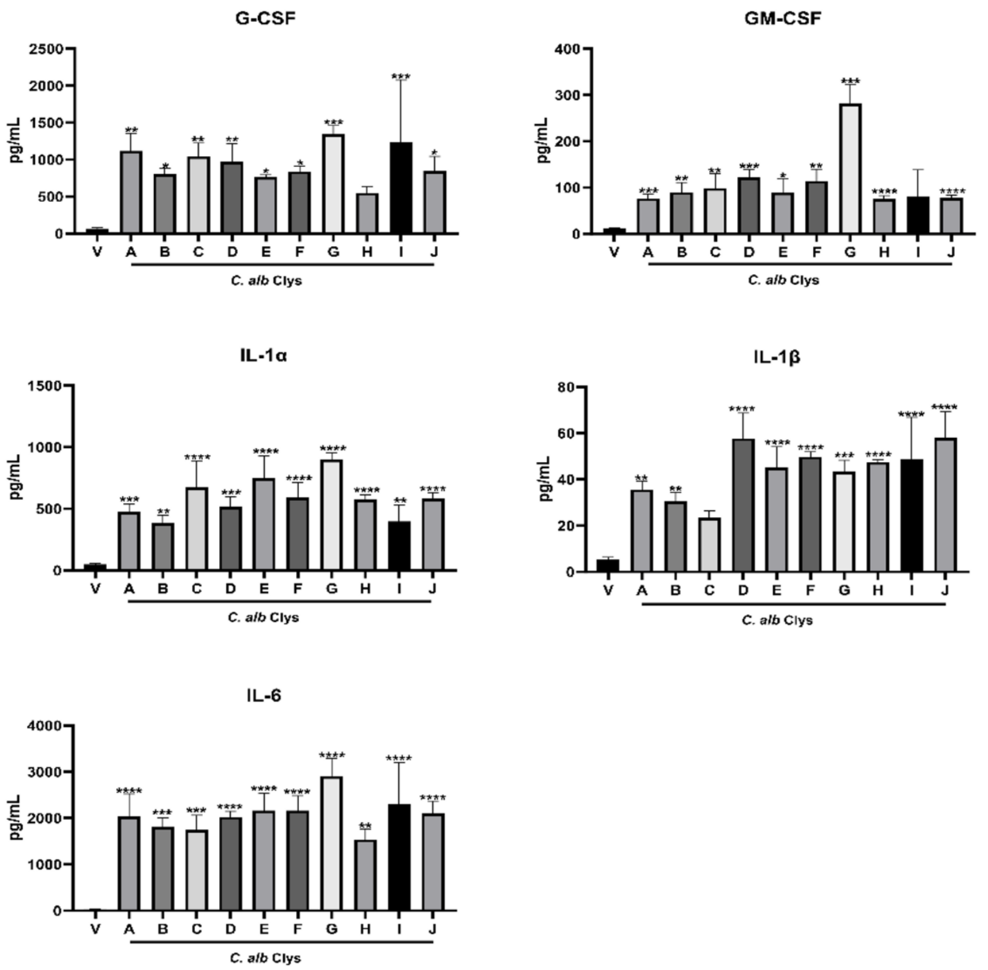

B

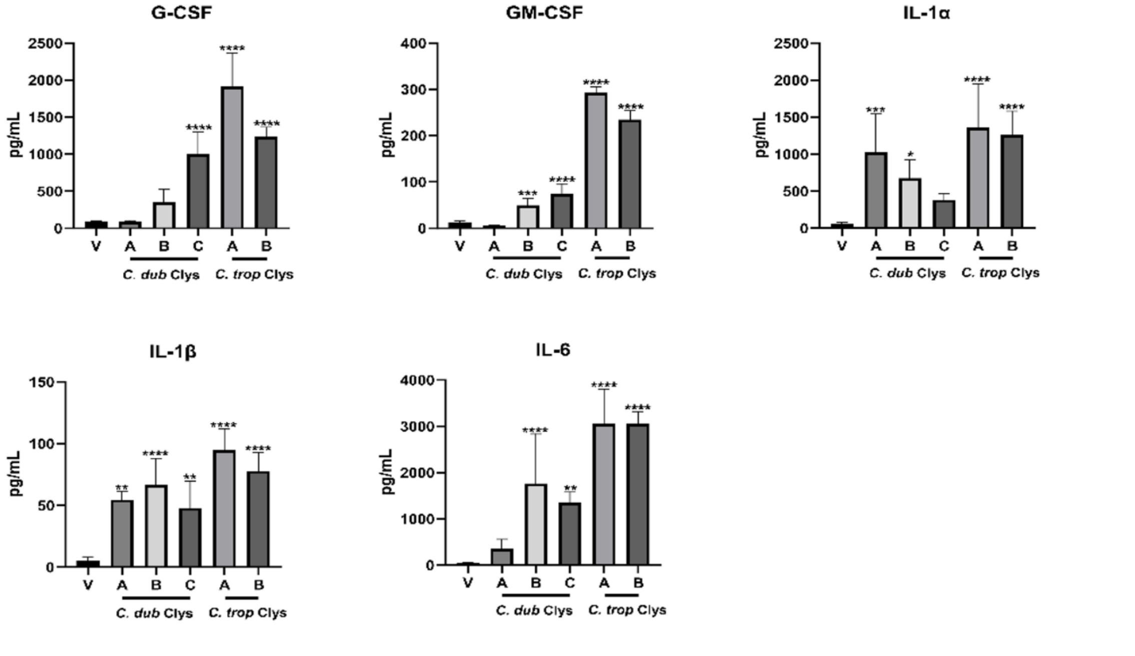

Supplemental Figure S9: Quantification of candidalysin-induced cytokine secretion. Quantification of G-CSF, GM-CSF, IL-1α, IL-1β and IL-6 secreted from TR146 epithelial cells

treated with 70 µM of each candidalysin variant from **(A)** *C. albicans*, and **(B)** *C. dubliniensis* and *C. tropicalis* for 24 h. Data are the mean + SD of n = 3 biological repeats. Statistical analysis was applied relative to vehicle-treated cells using a one-way ANOVA with a post hoc Bonferroni multiple comparison test; * $P < 0.05$, ** $P < 0.01$, *** $P < 0.001$, and P **** < 0.0001.

Supplemental Table S1. *C. dubliniensis* isolates investigated in the current study.

Isolate	Source	Country of origin	MLST clade	ITS genotype	Sequencing depth	*ECE1* nt 211	*ECE1* nt 231	*ECE1* nt236	Candidalysin variant	Reference
**CD36**	HIV positive	Ireland	1	1	99.6	GG	CC	CC	SIIGILTAILNNVPQIINVITTIIKSITGNKR	1
**Wü284**	HIV positive	Germany	1	1	103.1	GG	CC	CC	SIIGILTAILNNVPQIINVITTIIKSITGNKR	2
**AV5**	Avian excrement	Ireland	1	1	108	GG	CC	CC	SIIGILTAILNNVPQIINVITTIIKSITGNKR	3
**Is30**	Cancer, COPD	Iceland	1	1	86	GG	CC	CC	SIIGILTAILNNVPQIINVITTIIKSITGNKR	4
**CD06037**	Cystic Fibrosis	Ireland	2	2	254.7	AA	CC	CC	SIIGILTAILNNIPQIINVITTIIKSITGNKR	5
**Can4**	HIV positive	Canada	2	2	106	AA	CC	CC	SIIGILTAILNNIPQIINVITTIIKSITGNKR	6
**CD514**	HIV negative	Ireland	3	3	113	GG	CC	CC	SIIGILTAILNNVPQIINVITTIIKSITGNKR	7
**p7276**	HIV negative	Israel	3	3	90	GG	TT*	TT	SIIGILTAILNNVPQIINVIMTIIKSITGNKR	8
**Eg207**	Diabetes	Egypt	3	4	88	GG	CC	TT	SIIGILTAILNNVPQIINVIMTIIKSITGNKR	9
**p7718**	HIV negative	Israel	3	4	77.4	GG	CC	CC	SIIGILTAILNNVPQIINVITTIIKSITGNKR	7

Abbreviations: HIV, Human Immunodeficiency Virus; COPD, Chronic Obstructive Pulmonary Disease.
* Although the TT SNP in p7276 differs from the nucleotide sequence of CD36 (CC), amino acid identity is maintained (V).

REFERENCES

(1) Sullivan, D. J.; Westerneng, T. J.; Haynes, K. A.; Bennett, D. E.; Coleman, D. C. Candida Dubliniensis Sp. Nov.: Phenotypic and Molecular Characterization of a Novel Species Associated with Oral Candidosis in HIV-Infected Individuals. *Microbiology (Reading)* **1995**, *141 (Pt 7)*, 1507–1521. https://doi.org/10.1099/13500872-141-7-1507.

(2) Morschhäuser, J.; Ruhnke, M.; Michel, S.; Hacker, J. Identification of CARE-2-Negative Candida Albicans Isolates as Candida Dubliniensis. *Mycoses* **1999**, *42* (1–2), 29–32. https://doi.org/10.1046/j.1439-0507.1999.00259.x.

(3) McManus, B. A.; Sullivan, D. J.; Moran, G. P.; d'Enfert, C.; Bougnoux, M.-E.; Nunn, M. A.; Coleman, D. C. Genetic Differences between Avian and Human Isolates of Candida Dubliniensis. *Emerg Infect Dis* **2009**, *15* (9), 1467–1470. https://doi.org/10.3201/eid1509.081660.

(4) Asmundsdóttir, L. R.; Erlendsdóttir, H.; Agnarsson, B. A.; Gottfredsson, M. The Importance of Strain Variation in Virulence of Candida Dubliniensis and Candida Albicans: Results of a Blinded Histopathological Study of Invasive Candidiasis. *Clin Microbiol Infect* **2009**, *15* (6), 576–585. https://doi.org/10.1111/j.1469-0691.2009.02840.x.
(5) McManus, B. A.; Coleman, D. C.; Moran, G.; Pinjon, E.; Diogo, D.; Bougnoux, M.-E.; Borecká-Melkusova, S.; Bujdákova, H.; Murphy, P.; d'Enfert, C.; Sullivan, D. J. Multilocus Sequence Typing Reveals That the Population Structure of Candida Dubliniensis Is Significantly Less Divergent than That of Candida Albicans. *J Clin Microbiol* **2008**, *46* (2), 652–664. https://doi.org/10.1128/JCM.01574-07.
(6) Pinjon, E.; Sullivan, D.; Salkin, I.; Shanley, D.; Coleman, D. Simple, Inexpensive, Reliable Method for Differentiation of Candida Dubliniensis from Candida Albicans. *J Clin Microbiol* **1998**, *36* (7), 2093–2095. https://doi.org/10.1128/JCM.36.7.2093-2095.1998.
(7) Gee, S. F.; Joly, S.; Soll, D. R.; Meis, J. F. G. M.; Verweij, P. E.; Polacheck, I.; Sullivan, D. J.; Coleman, D. C. Identification of Four Distinct Genotypes of Candida Dubliniensis and Detection of Microevolution In Vitro and In Vivo. *J Clin Microbiol* **2002**, *40* (2), 556–574. https://doi.org/10.1128/JCM.40.2.556-574.2002.
(8) Polacheck, I.; Strahilevitz, J.; Sullivan, D.; Donnelly, S.; Salkin, I. F.; Coleman, D. C. Recovery of Candida Dubliniensis from Non-Human Immunodeficiency Virus-Infected Patients in Israel. *J Clin Microbiol* **2000**, *38* (1), 170–174.
(9) Al Mosaid, A.; Sullivan, D. J.; Polacheck, I.; Shaheen, F. A.; Soliman, O.; Al Hedaithy, S.; Al Thawad, S.; Kabadaya, M.; Coleman, D. C. Novel 5-Flucytosine-Resistant Clade of Candida Dubliniensis from Saudi Arabia and Egypt Identified by Cd25 Fingerprinting. *J Clin Microbiol* **2005**, *43* (8), 4026–4036. https://doi.org/10.1128/JCM.43.8.4026-4036.2005.
